# Association between clinically recorded alcohol consumption and initial presentation of 12 cardiovascular diseases: population based cohort study using linked health records

**DOI:** 10.1136/bmj.j909

**Published:** 2017-08-02

**Authors:** Steven Bell, Marina Daskalopoulou, Eleni Rapsomaniki, Julie George, Annie Britton, Martin Bobak, Juan P Casas, Caroline E Dale, Spiros Denaxas, Anoop D Shah, Harry Hemingway

**Affiliations:** 1 Department of Public Health and Primary Care, University of Cambridge, Strangeways Research Laboratory, Cambridge CB1 8RN, UK; 2 Research Department of Epidemiology and Public Health, University College London, London WC1E 7HB, UK; 3 Department of Infection and Population Health, University College London, Royal Free Hospital, London NW3 2PF, UK; 4 Farr Institute of Health Informatics Research (London), University College London, London NW1 2DA, UK

## Abstract

**Objectives** To investigate the association between alcohol consumption and cardiovascular disease at higher resolution by examining the initial lifetime presentation of 12 cardiac, cerebrovascular, abdominal, or peripheral vascular diseases among five categories of consumption.

**Design** Population based cohort study of linked electronic health records covering primary care, hospital admissions, and mortality in 1997-2010 (median follow-up six years).

**Setting** CALIBER (ClinicAl research using LInked Bespoke studies and Electronic health Records).

**Participants** 1 937 360 adults (51% women), aged ≥30 who were free from cardiovascular disease at baseline.

**Main outcome measures** 12 common symptomatic manifestations of cardiovascular disease, including chronic stable angina, unstable angina, acute myocardial infarction, unheralded coronary heart disease death, heart failure, sudden coronary death/cardiac arrest, transient ischaemic attack, ischaemic stroke, intracerebral and subarachnoid haemorrhage, peripheral arterial disease, and abdominal aortic aneurysm.

**Results** 114 859 individuals received an incident cardiovascular diagnosis during follow-up. Non-drinking was associated with an increased risk of unstable angina (hazard ratio 1.33, 95% confidence interval 1.21 to 1.45), myocardial infarction (1.32, 1.24 to1.41), unheralded coronary death (1.56, 1.38 to 1.76), heart failure (1.24, 1.11 to 1.38), ischaemic stroke (1.12, 1.01 to 1.24), peripheral arterial disease (1.22, 1.13 to 1.32), and abdominal aortic aneurysm (1.32, 1.17 to 1.49) compared with moderate drinking (consumption within contemporaneous UK weekly/daily guidelines of 21/3 and 14/2 units for men and women, respectively). Heavy drinking (exceeding guidelines) conferred an increased risk of presenting with unheralded coronary death (1.21, 1.08 to 1.35), heart failure (1.22, 1.08 to 1.37), cardiac arrest (1.50, 1.26 to 1.77), transient ischaemic attack (1.11, 1.02 to 1.37), ischaemic stroke (1.33, 1.09 to 1.63), intracerebral haemorrhage (1.37, 1.16 to 1.62), and peripheral arterial disease (1.35; 1.23 to 1.48), but a lower risk of myocardial infarction (0.88, 0.79 to 1.00) or stable angina (0.93, 0.86 to 1.00).

**Conclusions** Heterogeneous associations exist between level of alcohol consumption and the initial presentation of cardiovascular diseases. This has implications for counselling patients, public health communication, and clinical research, suggesting a more nuanced approach to the role of alcohol in prevention of cardiovascular disease is necessary.

**Registration** clinicaltrails.gov (NCT01864031).

## Introduction

The relation between alcohol consumption and cardiovascular disease is both complex and controversial.^1^ ^2^ There have been multiple systematic reviews and meta-analyses of the association between consumption and aggregated cardiovascular disease,^1^ ^2^ ^3^ ^4^ ^5^ ^6^ ^7^ as well as cardiovascular traits.^1^ ^8^ ^9^ Most have shown that, compared with non-drinking, moderate levels of alcohol intake are associated with a lower risk of morbidity and mortality from cardiovascular disease, as well as more favourable cardiovascular health profiles in general. There is, however, a growing scepticism around this observation, with recent commentary pieces pointing out several methodological shortcomings in the evidence on which the U shape is based.^10^ ^11^ ^12^ These include failure to have disaggregated the current non-drinking group into lifelong abstainers, former drinkers, and those who drink on an occasional basis. It is known that former drinkers (who might have quit for health reasons) have an increased risk of mortality from cardiovascular disease^13^ compared with lifelong non-drinkers; therefore combination of these two groups is likely to lead to the overestimation of the protective effects of moderate drinking. Similarly, it has been shown that the onset of ill health is associated with a reduction in regular consumption to drinking on an occasional basis,^14^ therefore combination of these individuals with non-drinkers also introduces bias.

Evidence from short term alcohol feeding interventions has shown that moderate drinking is related to higher concentrations of high density lipoprotein cholesterol and adiponectin, as well as lower concentrations of fibrinogen, but not other intermediate cardiovascular traits such as triglycerides.^8^ Given this, it could be hypothesised that moderate alcohol consumption might be protective for some cardiovascular diseases but not others.^15^ Similarly, there are concerns about residual confounding in moderate drinkers, and exploration of heterogeneity in the association between alcohol intake and subtypes of cardiovascular disease with different aetiology could help to alleviate part of this (for example, finding moderate drinking is associated with a lower risk of one cardiovascular disease but not another).

In an era of precision medicine, more detailed disease phenotype models are required to improve risk prediction at an individual and population level as well as be able to offer tailored advice to patients,^16^ and for this reason there have been calls for research into the association between alcohol consumption and deeper phenotypes of cardiovascular disease.^17^ The evidence base for specific phenotypes, however, is sparse compared with that of aggregated outcomes. Table A in the appendix provides an overview of research from major investigator led prospective observational studies as well as meta-analyses of the topic of alcohol consumption and a selection of specific cardiovascular diseases. Most research has focused on acute myocardial infarction or stroke (total and broad categories of ischaemic or haemorrhagic), which currently represent about 40% of incident cardiovascular events in the UK. Far less attention has been paid to other cardiovascular endpoints such as heart failure, cardiac arrest/sudden cardiac death, angina, peripheral arterial disease, subtypes of haemorrhagic stroke (intracerebral and subarachnoid haemorrhage), abdominal aortic aneurysm, and transient ischaemic attack, which collectively make up a substantial proportion of morbidity and healthcare expenditure in current clinical practice.^18^ ^19^

Few studies, however, have been sufficiently powered to examine individual cardiovascular diseases, and fewer still are in a position whereby they are also able to disaggregate the group of current non-drinker into non-drinkers, former drinkers, and occasional drinkers. Linked electronic health record data can be re-used to create cohorts of sufficient size and of satisfactory clinical resolution to be able to carry out such research.^18^ ^20^ Studies using linked electronic health record data in the context of cardiovascular disease have shown heterogeneous associations between disease phenotypes and various exposures, including sex, blood pressure, type 2 diabetes, and smoking.^21^ ^22^ ^23^ ^24^ ^25^ ^26^ ^27^ ^28^

We used linked electronic health record data to create a contemporary cohort with a median of six years of follow-up (11 637 926 person years) to investigate for the first time at large scale and within the same study whether the association with alcohol consumption differs across a wide range of incident cardiovascular diseases that are recognised to have different biological mediators. In addition to increased endpoint resolution, we also separated non-drinkers from former and occasional drinkers to provide to additional clarity in this debate.

## Methods

### Study design and participants

We included 1 937 360 anonymised patients from the CALIBER (CArdiovascular research using LInked Bespoke studies and Electronic health Records) programme.^29^ Details of the enrolment, follow-up, and data sources are presented in the appendix. Briefly, the cohort used patient data from the Clinical Practice Research Datalink (CPRD), comprising anonymised patient records from general practices in England. Patients were included if they were aged ≥30 from 1 January 1997 to 25 March 2010 and had no record indicating any cardiovascular disease before study entry (fig 1). CPRD provides primary care data on health behaviours, diagnoses, investigations, procedures, and prescriptions; and its accuracy and completeness are regularly audited. CPRD patients are representative of the UK population in terms of age, sex, ethnicity,^30^ ^31^ and overall mortality^32^ and have been validated for epidemiological research.^33^ Patient CPRD data were further linked with three other data sources: the Myocardial Ischaemia National Audit Project registry (MINAP)^34^; hospital episodes statistics (HES); and the Office for National Statistics (ONS). MINAP is a national registry of patients admitted to hospital with acute coronary syndromes in England and Wales. HES provides information on all hospital admissions and ONS on cause specific mortality records for all deaths in England and Wales. Information is coded with the hierarchical clinical coding schemes (Read codes,^35^ ICD-10 (international statistical classification of diseases, 10th revision), and Office of the Population Censuses and Surveys classification of interventions and procedures^36^). Our study protocol was registered with ClinicalTrials.gov (NCT01864031) before data were released to the lead author.

**Figure f1:**
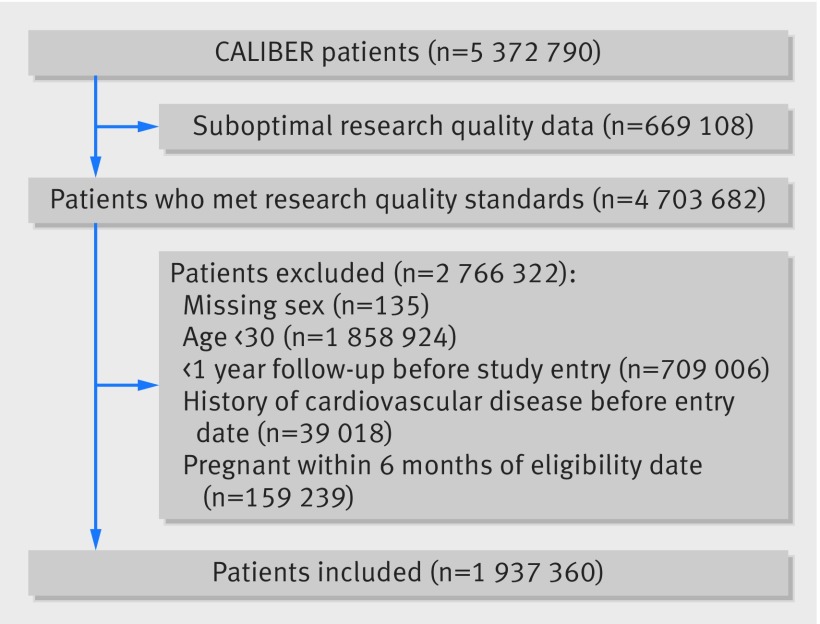
**Fig 1** Inclusion of patients in study of clinically recorded alcohol consumption and initial presentation of 12 cardiovascular diseases

### Alcohol assessment

General practitioners or practice nurses prospectively collected and coded self reported alcohol consumption on the consultation date in CPRD. We used the most recent record of alcohol consumption in the five years before entry into the study to classify participants’ drinking behaviour. In light of current debates on the U or J shaped relation observed between consumption and aggregated cardiovascular disease outcomes we defined five categories of drinking: non-drinkers (Read codes such as “teetotaller” and “non-drinker”), former drinkers (those with codes for “stopped drinking alcohol” and/or “ex-drinker”), occasional drinkers (those with codes for “drinks rarely” and/or “drinks occasionally”), current moderate drinkers (codes such as “alcohol intake within recommended sensible limits” and “light drinker”), and heavy drinkers (codes including “alcohol intake above recommended sensible drinking limits” and “hazardous alcohol use”). We also used data fields with information entered on daily and/or weekly amount of alcohol consumed to define participants as non-drinkers, moderate drinkers (drank within daily and/or weekly recommended sensible drinking limits for the UK at the time of observation^37^), and heavy drinkers (exceeded daily and/or weekly sensible drinking limits). We reclassified non-drinkers as former drinkers if they had any record of drinking or a history of alcohol abuse in their entire clinical record entered on CPRD before study entry. Further details, including a diagram depicting our coding scheme (fig B) plus a full list of the exact Read codes used to define drinking categories (table B) as well as a series of proof of concept validation analyses of the association between these groups and cardiovascular traits (fig C), are available in the appendix.

### Study endpoints

Patients were followed up until the date of an initial presentation of one of our cardiovascular endpoints (or death from non-cardiovascular causes) or were censored on the date they left the practice or the date of last data submission from their practice. We defined multiple endpoints on the basis of the first recorded diagnosis of one of****12 of the most common symptomatic manifestations of cardiovascular disease, including chronic stable angina, unstable angina, myocardial infarction, unheralded death from coronary heart disease, heart failure, cardiac arrest/sudden coronary death, transient ischaemic attack, ischaemic stroke, intracerebral haemorrhage, subarachnoid haemorrhage, peripheral arterial disease, and abdominal aortic aneurysm. We additionally estimated associations with non-cardiovascular disease mortality as well as coronary heart disease and stroke events that were not otherwise specified.

#### Secondary outcomes

For comparisons with existing studies we estimated models for aggregated coronary heart disease (myocardial infarction and unheralded death from coronary heart disease), cardiovascular disease (all cardiovascular endpoints other than stable angina), fatal cardiovascular disease (combination of fatal coronary heart disease and fatal cardiovascular disease), and all cause mortality. We also decomposed the myocardial infarction category into ST elevation, non-ST elevation, and myocardial infarction not otherwise specified. For further details see table C in the appendix.

### Covariates

Covariates considered in analyses included age (and age^2^), sex, area based socioeconomic deprivation (index of multiple deprivation^38^), smoking status, diabetes status, systolic blood pressure, body mass index (BMI), high density lipoprotein cholesterol, use of anti-hypertensive drugs or statins, and whether the patient had received dietary advice. Baseline covariates were defined with the most recent measurement up to one year before study entry (except smoking, which was up to three years). Additional information on how covariates were derived can be found on the CALIBER portal (www.caliberresearch.org/portal) and elsewhere.^21^ ^22^ ^23^ ^24^ ^26^

### Statistical analysis

We used multivariable Cox proportional hazard models to calculate hazard ratios and associated 95% confidence intervals for the association between categories of drinking and the initial presentation with specific cardiovascular disease phenotypes within a competing risk framework (that is, people could experience only one initial presentation). We plotted Schoenfeld residuals to ascertain that the proportional hazards assumption had not been violated. In our primary analysis we adjusted for age, socioeconomic deprivation, and smoking status. The baseline hazard function of each model was stratified by general practice and sex. Missing data were handled with multiple imputation^39^ under a missing at random assumption, and we carried out a series of sensitivity analyses adjusting for additional covariates, comparing imputed data (main analysis) to complete case data (n=1 104 838, with over a million participants also having information on smoking status), limiting analyses to different data sources, and using data only from 2004 onwards when recording of alcohol in primary care was incentivised. We also examined the association between alcohol categories and different cardiovascular diseases within subgroups defined by smoking status and BMI in a series of post hoc analyses suggested by reviewers. Further information on all sensitivity analyses is available in the appendix. Assuming mutual independence between endpoints, we assessed heterogeneity in associations across cardiovascular disease phenotypes within drinking categories using the I^2^ statistic. Our reference category in all models was moderate drinkers.^40^ All analyses were conducted with Stata v14.

### Patient involvement

No patients were involved in setting the research question or the outcome measures, nor were they involved in developing plans for design or implementation of the study. No patients were asked to advise on interpretation or writing up of results. There are no plans to disseminate the results of the research to study participants or the relevant patient community.

## Results

### Participant characteristics

Table 1 shows characteristics of the sample by category of drinking. Most study participants were non-smokers, had a BMI within the normal range, and were free from diabetes. All types of current non-drinkers were more likely to belong to the most deprived socioeconomic fifth. The distribution of 114 859 initial presentations across a broad range of cardiovascular disease endpoints within each drinking category is shown in fig D in the appendix.

**Table 1 tbl1:** Baseline demographic and health related characteristics of 1 937 360 adults according to clinically recorded drinking category. Figures are percentages^*^ unless stated otherwise

	**Non-drinker (14.3%)**	**Former drinker (3.7%)**	**Occasional drinker (11.9%)**	**Moderate drinker (61.7%)**	**Heavy drinker (8.4%)**	**Alcohol status missing**	**Total**
Mean (SD) age (years)	48.5 (16.6)	49.5 (16.6)	48.1 (15.7)	45.8 (14.2)	45.8 (12.7)	48.0 (16.1)	47.1 (15.4)
Men	33.1	37.3	33.5	49.8	66.9	53.5	49.5
Women	66.9	62.7	66.5	50.2	33.1	46.5	50.5
Most deprived 5th of socioeconomic deprivation	30.6	28.9	25.1	15.7	20.5	20.1	20.0
Smoking status:							
Non-smoker	72.3	49.5	62.0	58.9	39.4	73.8	63.5
Former smoker	10.2	20.7	15.9	18.7	21.2	13.3	16.2
Current smoker	17.5	29.8	22.1	22.4	39.5	12.9	20.3
Systolic blood pressure (mm Hg)	129.3 (19.0)	130.5 (18.2)	129.9 (18.2)	129.3 (17.0)	133.5 (17.1)	133.7 (18.9)	131.0 (18.1)
Categories of BMI:							
Underweight (<18.5)	3.2	3.2	2.1	1.7	1.8	2.7	2.1
Normal weight (18.5-24)	41.8	39.5	40.5	45	41.2	39.4	43.0
Overweight (25-29)	32.3	32.3	33.8	35.9	38.6	32.4	34.9
Moderately obese (30-34)	19.8	21.6	20.6	16	17.1	22	17.9
Morbidly obese (≥35)	2.9	3.4	2.9	1.5	1.1	3.5	2.1
Diabetes	5.1	6.7	3.7	2.4	2.2	1.9	2.6
Median (IQR) HDLC concentration (mmol/L)	1.3 (1.1-1.5)	1.2 (1.0-1.5)	1.3 (1.1-1.6)	1.3 (1.1-1.6)	1.4 (1.2-1.8)	1.3 (1.1-1.6)	1.3 (1.1-1.6)
Used anti-hypertensive drugs	19.7	26.6	21.1	16.1	17.2	15.1	16.6
Used statins	4.4	7.0	3.9	3.0	3.4	1.3	2.5
Offered dietary advice	45.9	58.7	53.8	47.9	45.6	9.6	31.8

BMI=body mass index; IQR=interquartile range; HDLC=high density lipoprotein cholesterol.

*Row percentages displayed for drinking categories calculated only within those with information on alcohol consumption (n=1 104 838; 57% of overall sample). In imputed data, drinking category proportions are as follows: 14.7% non-drinkers, 3.2% former drinkers, 11.6% occasional drinkers, 62.4% moderate drinkers, and 8.1% heavy drinkers. Proportion of participants with non-missing values of covariates: smoking 73.0% (1 413 749 participants), systolic blood pressure 73.2% (1 418 578 participants), BMI 30.6% (592 127 participants), and HDLC 5.5% (107 080 participants). All other covariates have 100% coverage.

### Outcomes for common aggregated cardiovascular disease endpoints and all cause mortality

Figure 2 shows the association between categories of clinically recorded alcohol consumption and coronary heart disease, cardiovascular disease, fatal cardiovascular disease, and all cause mortality. We observed classic J shaped associations for cardiovascular disease (all and fatal) and all cause mortality, with non-drinkers, former drinkers, and heavy drinkers having an increased risk compared with moderate drinkers. For coronary heart disease, though we found that non-drinkers had an increased risk of experiencing an event (hazard ratio 1.31, 95% confidence interval 1.27 to 1.36), we observed no difference in risk in heavy drinkers (0.97, 0.90 to 1.06) compared with moderate drinkers.

**Figure f2:**
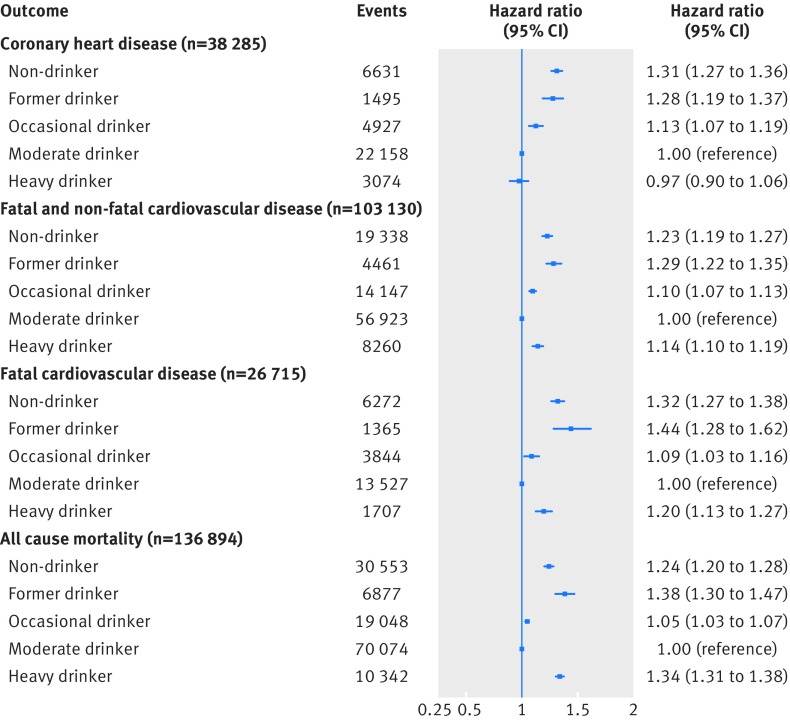
**Fig 2** Multivariable adjusted hazard ratios for aggregated cardiovascular endpoints for clinically recorded non-drinkers and former, occasional, and heavy drinkers compared with moderate drinkers in cohort of 1.93 million adults adjusted for age (and age^2^), sex, socioeconomic deprivation, and smoking status

### Outcomes for specific phenotypes of cardiovascular disease

Figures 3 and 4[Fig f3 f4] show findings from multivariable Cox models for cardiac and non-cardiac cardiovascular diseases, respectively. Compared with moderate drinkers, non-drinkers had an increased risk of developing unstable angina (hazard ratio 1.33, 95% confidence interval 1.21 to 1.45) or experiencing a myocardial infarction (1.32, 1.24 to 1.41), unheralded coronary death (1.56, 1.38 to 1.76), heart failure (1.24, 1.11 to 1.38), ischaemic stroke (1.12, 1.01 to 1.24), peripheral arterial disease (1.22, 1.13 to 1.32), and abdominal aortic aneurysm (1.32, 1.17 to 1.49) as their initial presentation of cardiovascular disease.

**Figure f3:**
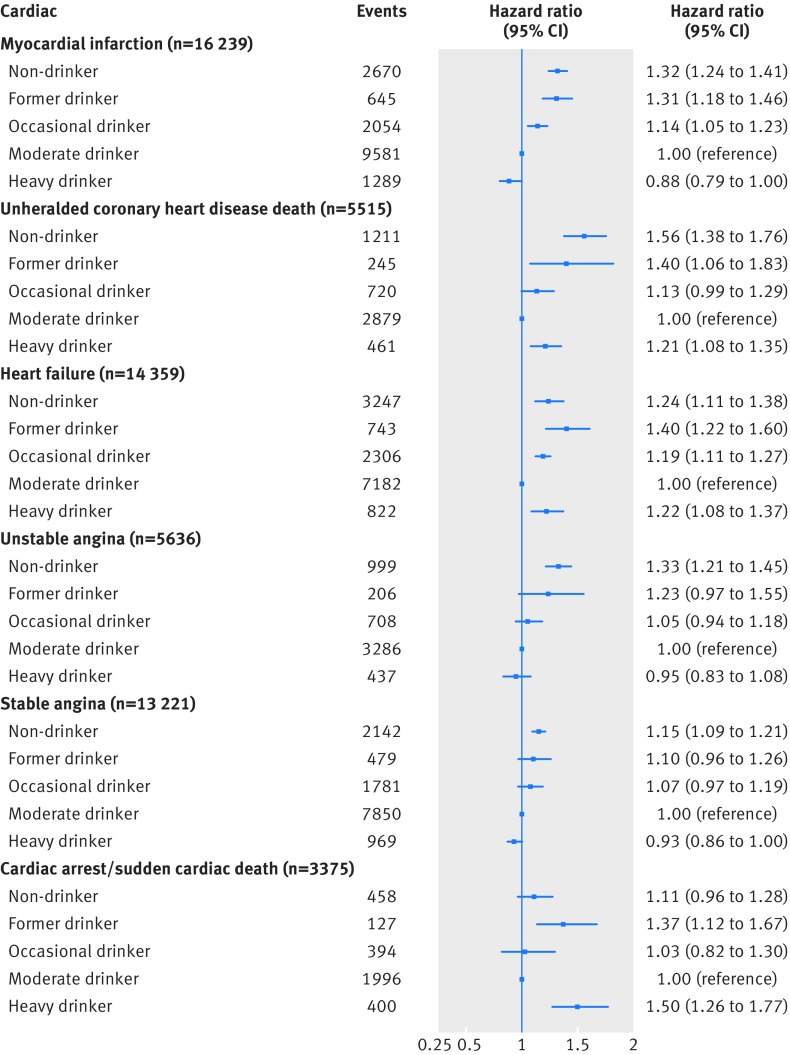
**Fig 3** Multivariable adjusted hazard ratios for cardiac cardiovascular diseases for clinically recorded non-drinkers and former, occasional, and heavy drinkers compared with moderate drinkers in cohort of 1.93 million adults adjusted for age (and age^2^), sex, socioeconomic deprivation, and smoking status

**Figure f4:**
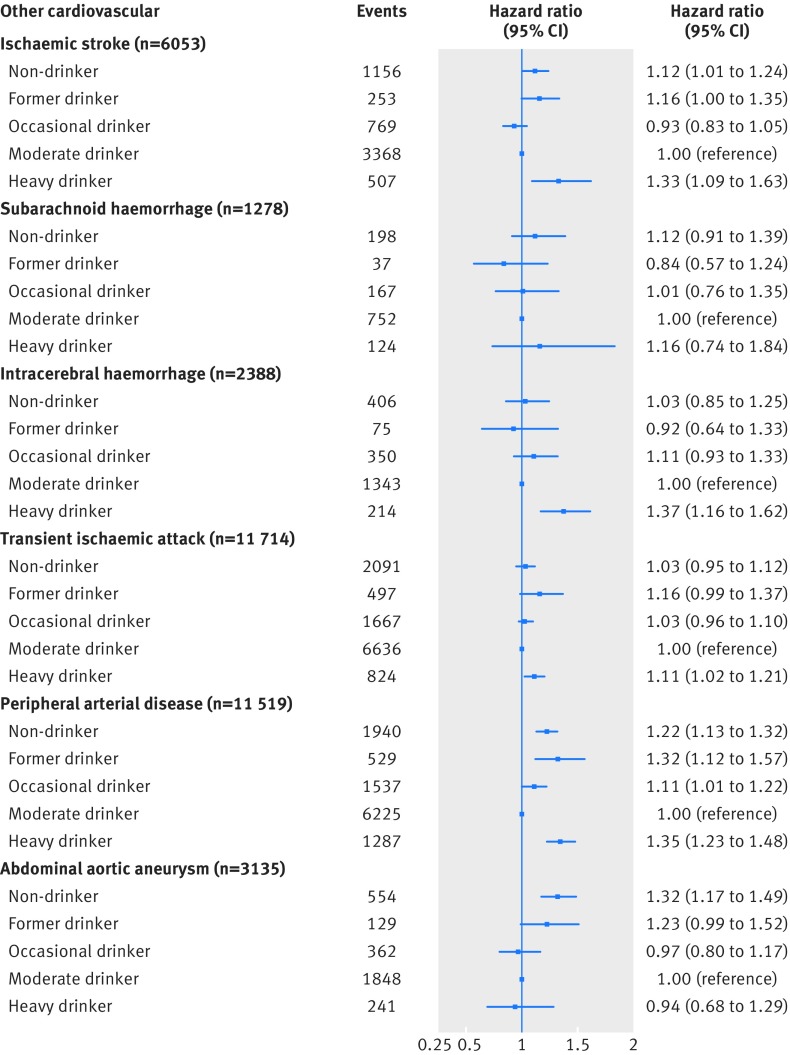
**Fig 4** Multivariable adjusted hazard ratios for non-cardiac cardiovascular diseases for clinically recorded non-drinkers and former, occasional, and heavy drinkers compared with moderate drinkers in cohort of 1.93 million adults adjusted for age (and age^2^), sex, socioeconomic deprivation, and smoking status

Heavy drinkers had an increased risk of their initial presentation of cardiovascular disease being unheralded coronary death (hazard ratio 1.21, 95% confidence interval 1.08 to 1.35), heart failure (1.22, 1.08 to 1.37), cardiac arrest/sudden coronary death (1.50, 1.26 to 1.77), and transient ischaemic attack (1.11, 1.02 to 1.21) (fig 3) and ischaemic stroke (1.33, 1.09 to 1.63), intracerebral haemorrhage (1.37, 1.16 to 1.62), and peripheral arterial disease (1.35, 1.23 to 1.48) (fig 4). Heavy drinkers, however, had a lower risk of experiencing a myocardial infarction (0.88, 0.79 to 1.00) and stable angina (0.93, 0.86 to 1.00) as their first cardiovascular disease (fig 3).

Former drinkers had an augmented risk of unstable angina (hazard ratio 1.23, 95% confidence interval 0.97 to 1.55), myocardial infarction (1.31, 1.18 to 1.46), unheralded coronary death (1.40, 1.06 to 1.83), heart failure (1.40, 1.22 to 1.60), and cardiac arrest/sudden coronary death (1.37, 1.12 to 1.67) (fig 3) and ischaemic stroke (1.16, 1.00 to 1.35), transient ischaemic attack (1.16, 0.99 to 1.37), peripheral arterial disease (1.32, 1.12 to 1.57), and abdominal aortic aneurysm (1.23, 0.99 to 1.52) (fig 4) being their initial cardiovascular disease presentation.

Occasional drinkers had an increased risk of myocardial infarction (hazard ratio 1.14, 95% confidence interval 1.05 to 1.23), unheralded coronary death (1.13, 0.99 to 1.29), and heart failure (1.19, 1.11 to 1.27) (fig 3) and peripheral arterial disease (1.11, 1.01 to 1.21) (fig 4).

We present findings for non-cardiovascular disease death (as well as coronary heart disease and stroke, not otherwise specified) in fig F in the appendix. All other categories of drinking were associated with an increased risk of non-cardiovascular disease mortality compared with moderate drinkers.

We found no significant heterogeneity in the association with alcohol consumption across subtypes of myocardial infarction (fig F in appendix). There was, however, evidence of significant heterogeneity in the initial presentation of cardiovascular diseases within the categories of non-drinking and heavy drinking (table D in appendix).

### Effect modification by sex

We found some evidence that the association between alcohol consumption and heart failure and non-cardiovascular disease mortality differed by sex (see table E and figs I and J in the appendix). Specifically, among women we observed no increased risk between heavy drinking and heart failure and an attenuated, although still increased, risk in women who did not drink compared with moderate drinkers.

### Sensitivity analyses

Findings of sensitivity analyses are in the appendix. Interpretation did not change substantially when we adjusted only for age and sex (fig G) or after additional adjustment for systolic blood pressure, BMI, diabetes mellitus, high density lipoprotein cholesterol, use of anti-hypertensive drugs or statins, and whether then patient had received dietary advice (fig H). Similar associations were observed when we restricted analyses to endpoints determined with secondary care and mortality data sources (fig K), as well as fatal events only (fig L). There were no notable differences in the associations we observed when we used data only from 2004 onwards (fig M). Our findings when we used complete case methods were broadly concordant with those obtained using multiple imputation (fig N).

### Post hoc analyses

Estimates from post hoc analyses within subgroups defined by smoking status (figs O and P) and BMI (figs Q, R, S) are also presented in the appendix. Our interpretation was not materially altered when we limited analyses to any specific subgroup. It is worth noting that as these analyses were restricted to observed data out of necessity, statistical power was noticeably reduced, and, while there were some differences between the point estimates in subgroups for certain endpoints (often rarer events), the confidence intervals often overlapped and included the point estimates (fig 3).^41^ Suggestive differences included that the lower risk of myocardial infarction in heavy drinkers was attenuated in current smokers (hazard ratio 0.95, 95% confidence interval 0.83 to 1.08) and those with a BMI in the normal range (1.00, 0.79 to 1.27). Non-drinking was not associated with an increased risk of cardiac arrest/sudden coronary death or abdominal aortic aneurysm in never smokers and those considered obese.

## Discussion

In this population based cohort study of a large scale contemporary clinical sample we found considerable heterogeneity in the association between recorded alcohol consumption and the initial presentation of 12 cardiovascular diseases.

Our findings for aggregated endpoints are in line with those of previous observational studies,^42^ ^43^ showing that there is an increased risk of coronary heart disease, cardiovascular disease, and all cause mortality in the group of non-drinkers from whom former and occasional drinkers have been removed. At the same time, compared with moderate drinkers, heavy drinkers have an increased risk of experiencing all but coronary heart disease. This lends further support to the validity of using routinely collected clinical data on alcohol consumption in research and risk prediction algorithms.

### Novel associations and improved resolution for association between alcohol consumption and specific cardiovascular diseases

Our finding that moderate alcohol consumption is associated with a lower risk of initially presenting with a range of cardiovascular diseases is consistent with results of previous smaller studies (see table A). We extend this earlier work in clarifying that the protective effect observed for moderate drinking and major clinical outcomes such as myocardial infarction,^43^ ischaemic stroke,^44^ sudden coronary death,^45^ ^46^ heart failure,^47^ peripheral arterial disease,^48^ and abdominal aortic aneurysm^49^ is present even after separation of the group of current non-drinkers into more specific categories. Unlike others,^50^ we found no evidence of a protective effect of moderate drinking for subarachnoid haemorrhage, but we observed an increased risk of intracerebral haemorrhage among heavy drinkers, which is consistent with reports elsewhere.^51^ ^52^ Other studies have also shown protective effects of alcohol consumption, even at heavy levels, for myocardial infarction.^43^ ^53^

In most outcomes for which we found a protective effect of moderate drinking, the risk of initially presenting with that endpoint was higher in former drinkers, which is consistent with the “sick quitter” hypothesis, although we still observed excess risk among non-drinkers.^10^ ^13^

To our knowledge, we have provided the first set of analyses examining the association between alcohol intake and subcategories of myocardial infarction, finding no heterogeneity across subtypes of ST elevation, non-ST elevation, and myocardial infarction not otherwise specified. This is also the first study to explore the association between not drinking and transient ischaemic attack.^54^ There was no difference in risk between non-drinking and occasional drinking groups compared with those with a moderate alcohol intake. Moderate drinking, however, was associated with a lower risk of initially presenting with stable angina in contrast with non-drinking .^55^ ^56^ Furthermore, we report the first findings for alcohol consumption and unheralded coronary death, an outcome of major importance to public health, showing that both non-drinkers and heavy drinkers were more likely than moderate drinkers to present with coronary death with no previous symptomatic presentations. We are also the first to show that heavy drinkers are more likely to initially present with peripheral arterial disease^55^ and fill in a current gap in the evidence base by showing no association between heavy drinking and the initial presentation of abdominal aortic aneurysm.^49^

### Sex differences in association between alcohol consumption and cardiovascular diseases

We observed few associations that differed in their magnitude by sex, which is consistent with a recent meta-analysis for aggregated cardiovascular disease.^57^ We extend this to multiple cardiovascular disease phenotypes (except heart failure, for which there was a significant sex difference).

### Strengths: high resolution of exposure and endpoints in a contemporary clinical cohort

One of the primary strengths of our study is its large size, which allowed us to examine risk of multiple cardiovascular diseases within the same sample, some of which are too rare to reliably investigate in smaller studies. Use of data from several electronic health record databases further improved the validity of our cardiovascular disease endpoints.^33^ Furthermore, we attempted to stratify the group of current non-drinkers into non-drinkers and former and occasional drinkers to clarify whether the observed protective effects of moderate drinking were present when these groups were separated from each other. Another strength of our use of a contemporary cohort is that our exposure variable reflects the drinking habits encountered by health workers in present day clinical practice, whereas the information used in consented cohort studies often echoes drinking behaviour prevalent 15-20 years or more ago. This strength also carries over to other behaviours and clinical practice.

### Limitations

Of course, our study is not without limitations. For example, our categories of drinking were based not only on self reported alcohol intake as reported by patients to their general practitioner or practice nurse but also our own judgement as how best to combine the recorded codes. Self reported measures of drinking have been criticised,^58^ and it is likely that a certain degree of misclassification bias is present in our drinking categories—for example, some of the occasional drinkers were probably regular/moderate drinkers while some moderate drinkers were likely to be heavy drinkers. Therefore, the heavy drinkers in our study could represent the more extreme end of the drinking spectrum.

Furthermore, no standard questions about drinking were used by all health professionals during the study period, meaning that their own personal biases might have resulted in further misclassification^59^—for example, whether they consume alcohol or not. Individuals might respond more honestly when recording their alcohol consumption on a paper questionnaire than directly to a medical professional (differential reporting by sex, fear of being judged, etc), but there was no way to quantify what proportion of data were collected by either method. It is important to bear in mind, however, that the information on alcohol consumption we used is intrinsically relevant to clinical practice as it was gathered as part of routine care and is therefore the sort of information on which clinicians will base their subsequent advice and/or treatment of patients in day to day clinical practice. Additionally, it has been standard practice in every meta-analysis of alcohol consumption and aggregated cardiovascular disease (plus other diseases) to date to combine data collected using different methods, and we do not consider our approach any more inherently biased than that. We also carried out a series of analyses linking the categories of drinking we used in this study to multiple cardiovascular traits, and they behaved as expected indicating acceptable validity of our approach.

We were unable to account for differences in risk by beverage type, though findings in this area are largely mixed,^43^ ^60^ ^61^ and it has been argued that beverage specific effects are more often a result of residual confounding by socioeconomic position^62^ than true effects. Furthermore, we were unable to account for the impact of drinking pattern^4^ ^43^ ^63^ or changes in drinking over time^64^ ^65^ ^66^ ^67^ on different cardiovascular outcomes. Frequency of consumption is an important omission as it is known that most people do not spread their drinking equally across the week and even isolated episodes of heavy drinking are enough to eliminate the protective effects observed for coronary heart disease in otherwise moderate drinkers.^4^ ^63^ We also did not explicitly seek to determine “thresholds” of drinking associated with the lowest risk of harm, instead we used existing clinically recorded data on alcohol consumption to examine for the first time at large scale and within the same study the association between broadly defined categories of drinking (with an emphasis placed on separating different non-drinking groups) and the initial presentation of a range of pathologically diverse cardiovascular diseases. After we have shown that heterogeneous associations exist across cardiovascular endpoints, a logical next step forward would be to more thoroughly investigate the shape of the dose-response association using continuous measures of alcohol consumption. Furthermore, our thorough examination of alcohol consumption recorded in electronic health records has additional clinical implications in having highlighted areas in which measurement of alcohol could be improved in clinical practice (such as drinking pattern).

While we examined a range of cardiovascular diseases, we were unable to resolve some specific subtypes—for example, thrombotic versus embolic ischaemic stroke. This means that an even greater degree of heterogeneity could be present across subtypes of disease. While we tried to minimise measurement error in the group of non-drinkers by using a patient’s entire clinical history to define them as former drinkers if they had any record of drinking, it is likely that this approach did not capture all former drinkers. As such it is possible that the increased risk of initially presenting with several cardiovascular diseases in non-drinkers is partly caused by drinking category contamination/existing comorbidities (for example, we found that non-drinkers were more likely to have diabetes or be obese). Finally, as with all observational studies, we were unable to exclude residual confounding—for example, we did not have information on amount of tobacco smoked (as well as other smoking related traits such as age at initiation, pattern/duration of smoking, and exposure to secondhand smoke), dietary habits, or level of physical activity as these are lacking in the pre-existing electronic databases we used. By assessing the relatively negligible changes in the magnitude of the effect estimates observed for alcohol and aggregated coronary heart disease,^63^ ischaemic stroke,^68^ and myocardial infarction,^43^ pre- and post-adjustment for dietary components and physical activity in large studies to date, however, we are somewhat confident that even if we were able to adjust for these factors, our overall conclusions would not materially change.

### Clinical and public health implications

As noted previously, there is growing belief that the cardiovascular benefits of moderate drinking might have been overestimated,^10^ including in a recent large scale Mendelian randomisation study^69^ that found no protective effects of moderate alcohol intake for aggregate cardiovascular disease (though there have been some critical commentaries of this study^70^ ^71^). A sister paper from the Alcohol-*ADH1B* consortium, however, found evidence for non-linear associations between alcohol intake and some cardiovascular disease traits, including non-high density lipoprotein cholesterol, BMI, waist circumference, and C reactive protein.^72^ We would expect these associations to then translate into greater heterogeneity of association with specific cardiovascular disease endpoints as seen here, underlying the importance of greater granularity in endpoint specification.

The main clinical implications stemming from our findings are concerned with primary prevention and personalised risk. For example, if a patient reports heavy drinking they can be informed that if they continue to do so they have an increased risk of initial presentation with****ischaemic stroke, heart failure, cardiac arrest, transient ischaemic attack, intracerebral haemorrhage, or peripheral arterial disease, as well coronary death with no previous symptoms. These findings could have further translational value in an era whereby risk prediction algorithms are being developed and/or improved for specific cardiovascular disease phenotypes through having shown that the association with alcohol consumption is not common across diseases. Similarly, having shown that data on clinically recorded alcohol consumption can be validly used in research settings, in the future such information could be incorporated in disease specific risk prediction algorithms nested in clinical practice.

While we did find that heavy drinkers had a lower risk of presenting with a myocardial infarction, this needs to be considered within the context of our study, which was focused on initial presentation. This does not mean that heavy drinkers will not go on to experience a myocardial infarction in the future, just that they were less likely to present with this as their first diagnosis compared with moderate drinkers. Furthermore, heavy drinkers were more likely to initially present with death from causes other than cardiovascular disease, meaning it is possible that they are less likely to initially present with any cardiovascular disease because they die from other causes before they are able to develop a cardiovascular disease.

Similarly, while we found that moderate drinkers were less likely to initially present with several cardiovascular diseases than non-drinkers, it could be argued that it would be unwise to encourage individuals to take up drinking as a means of lowering their risk (although it must be noted that the findings from this study do not directly support this as we did not consider transitions from non-drinking to drinking). This is because there are arguably safer and more effective ways of reducing cardiovascular risk, such as increasing physical activity^73^ ^74^ and smoking cessation,^75^ which do not incur increased risks of alcohol related harm such as alcohol dependence, liver disease, and cancer.^76^ ^77^ ^78^ It is also worth bearing in mind that our focus was on risk of initial presentation with one cardiovascular disease rather than another, not absolute risk of cardiovascular disease. Ultimately an individual’s decision to drink should not be considered in isolation from other health behaviours or risk factors and instead be motivated by their own personal circumstances.

Finally, from a public health perspective, our finding that moderate drinking is not universally associated with a lower risk of all cardiovascular conditions also supports the decision not to incorporate the apparent protective effects of drinking for cardiovascular disease in the recent UK chief medical officers’ alcohol guidelines review.^76^

### Conclusions

Collectively, our findings, from the most comprehensive study to date of the relation between alcohol consumption and risk of cardiovascular disease, indicate that moderate alcohol consumption is associated with a lower risk of initially presenting with several, but not all, cardiovascular diseases. Similarly, we show that heavy drinking is differentially associated with a range of such diseases. This has implications for patient counselling, public health communication, and disease prediction algorithms and suggests the necessity for a more nuanced approach to the role of alcohol consumption in the prevention of cardiovascular disease.

**What is already known on this topic**

Moderate alcohol consumption is thought to be associated with a lower risk of developing cardiovascular disease compared with abstinence or heavy drinking.
There are ongoing debates about the role of combining different types of current non-drinkers in producing this apparent protective effect. Specifically, former or occasional drinkers might have reduced or ceased drinking because of ill health, making the aggregated non-drinking group artificially seem to have a higher risk of cardiovascular disease and mortality
Less is known about the role of alcohol consumption in the aetiology of specific cardiovascular diseases; where studies exist they are often few in number, small in size, have combined different types of non-drinkers, and have not excluded all forms of cardiovascular disease before the primary event

**What this study adds**

This large scale study of 1.93 million adults without cardiovascular disease at baseline showed that moderate drinking is associated with a lower risk of initial presentation with several, but not all, cardiovascular diseases, even after separation of groups of non-drinkers
Though higher levels of alcohol intake are associated with a lower risk of initial presentation with myocardial infarction, this is offset by heavier drinkers having a greater risk of initially presenting with several other cardiovascular diseases as well as mortality from non-cardiovascular causes
Data on clinically recorded alcohol consumption can be validly used in research and practice

## Web Extra

Extra material supplied by the author

## Notes

Contributors: SB and HH conceived the research question with additional input from MD, ER, AB, and MB. JG, SD, and HH participated in development of coding algorithms, the CALIBER research platform, and study design. ADS assisted in defining endpoints and the development of coding algorithms for other variables. SD linked, managed, and provided the data. SB carried out the statistical analysis and wrote the first draft of the manuscript. All authors provided additional intellectual content, contributed to critical revisions of the manuscript, and read and approved the final submitted version. SB is guarantor.

Ethical approval: The study was part of the CALIBER programme, approved by the MINAP Academic Group and by the CPRD independent scientific advisory committee.

Data sharing: Electronic health records are, by definition, considered “sensitive” data in the UK by the Data Protection Act and cannot be shared via public deposition because of information governance restrictions in place to protect patient confidentiality. The CALIBER data portal is available for consultation online at www.caliberresearch.org/. Access to data for external researchers (not affiliated with CALIBER investigators) is provided within the CALIBER “safe haven” environment, which currently requires researchers to be physically based in either UCL (clinical epidemiology group) or the London School of Hygiene and Tropical Medicine (Liam Smeeth). Access to data is available only once approval has been obtained through the individual constituent entities controlling access to the data. The primary care data can be requested via application to the Clinical Practice Research Datalink (https://www.cprd.com); secondary care data can be requested via application to the hospital episode statistics from the UK Health and Social Care Information Centre (www.hscic.gov.uk/hesdata); myocardial infarction registry data are available by application to the National Institute for Cardiovascular Outcomes Research Myocardial Ischaemia National Audit Project (www.ucl.ac.uk/nicor/audits/minap); and mortality data are available by application to the UK Office for National Statistics (www.ons.gov.uk/ons/index.html). The phenotype algorithms described in this paper are available via the CALIBER website at www.caliberresearch.org.

Transparency: The lead author affirms that the manuscript is an honest, accurate, and transparent account of the study being reported; that no important aspects of the study have been omitted; and that any discrepancies from the study as planned have been explained.

## References

[1] Fernández-Solà J. Cardiovascular risks and benefits of moderate and heavy alcohol consumption. Nat Rev Cardiol 2015;12:576-87. 10.1038/nrcardio.2015.91 pmid:26099843. 26099843

[2] Mukamal KJ, Rimm EB. Alcohol consumption: risks and benefits. Curr Atheroscler Rep 2008;10:536-43. 10.1007/s11883-008-0083-2 pmid:18937903. 18937903

[3] Roerecke M, Rehm J. The cardioprotective association of average alcohol consumption and ischaemic heart disease: a systematic review and meta-analysis. Addiction 2012;107:1246-60. 10.1111/j.1360-0443.2012.03780.x pmid:22229788. 22229788 PMC3348338

[4] Roerecke M, Rehm J. Alcohol consumption, drinking patterns, and ischemic heart disease: a narrative review of meta-analyses and a systematic review and meta-analysis of the impact of heavy drinking occasions on risk for moderate drinkers. BMC Med 2014;12:182 10.1186/s12916-014-0182-6 pmid:25567363. 25567363 PMC4203905

[5] Ronksley PE, Brien SE, Turner BJ, Mukamal KJ, Ghali WA. Association of alcohol consumption with selected cardiovascular disease outcomes: a systematic review and meta-analysis. BMJ 2011;342:d671 10.1136/bmj.d671 pmid:21343207. 21343207 PMC3043109

[6] Movva R, Figueredo VM. Alcohol and the heart: to abstain or not to abstain?Int J Cardiol 2013;164:267-76. 10.1016/j.ijcard.2012.01.030 pmid:22336255. 22336255

[7] O’Keefe JH, Bybee KA, Lavie CJ. Alcohol and cardiovascular health: the razor-sharp double-edged sword. J Am Coll Cardiol 2007;50:1009-14. 10.1016/j.jacc.2007.04.089 pmid:17825708. 17825708

[8] Brien SE, Ronksley PE, Turner BJ, Mukamal KJ, Ghali WA. Effect of alcohol consumption on biological markers associated with risk of coronary heart disease: systematic review and meta-analysis of interventional studies. BMJ 2011;342:d636 10.1136/bmj.d636 pmid:21343206. 21343206 PMC3043110

[9] Mathews MJ, Liebenberg L, Mathews EH. The mechanism by which moderate alcohol consumption influences coronary heart disease. Nutr J 2015;14:33 10.1186/s12937-015-0011-6 pmid:25889723. 25889723 PMC4389579

[10] Chikritzhs T, Stockwell T, Naimi T, Andreasson S, Dangardt F, Liang W. Has the leaning tower of presumed health benefits from ‘moderate’ alcohol use finally collapsed?Addiction 2015;110:726-7. 10.1111/add.12828 pmid:25613200. 25613200

[11] Fekjaer HO. Alcohol-a universal preventive agent? A critical analysis. Addiction 2013;108:2051-7. 10.1111/add.12104 pmid:23297738. 23297738

[12] Stockwell T, Greer A, Fillmore K, Chikritzhs T, Zeisser C. How good is the science?BMJ 2012;344:e2276 , author reply e2294. 10.1136/bmj.e2276 pmid:22453883. 22453883

[13] Roerecke M, Rehm J. Ischemic heart disease mortality and morbidity rates in former drinkers: a meta-analysis. Am J Epidemiol 2011;173:245-58. 10.1093/aje/kwq364 pmid:21156750. 21156750 PMC3105267

[14] Ng Fat L, Cable N, Shelton N. Worsening of health and a cessation or reduction in alcohol consumption to special occasion drinking across three decades of the life course. Alcohol Clin Exp Res 2015;39:166-74. 10.1111/acer.12596 pmid:25623415. 25623415 PMC4329335

[15] Mukamal K. Alcohol intake and noncoronary cardiovascular diseases. Ann Epidemiol 2007;17(Suppl):S8-12. 10.1016/j.annepidem.2007.01.003 pmid:17478332. 17478332 PMC4446120

[16] Delude CM. Deep phenotyping: The details of disease. Nature 2015;527:S14-5. 10.1038/527S14a pmid:26536218. 26536218

[17] Breslow RA, Mukamal KJ. Measuring the burden--current and future research trends: results from the NIAAA Expert Panel on Alcohol and Chronic Disease Epidemiology. Alcohol Res 2013;35:250-9.pmid:24881334. 24881334 10.35946/arcr.v35.2.16 PMC3908717

[18] Newby LK. Understanding Population Cardiovascular Health: Harnessing the Power of Electronic Health Records. Circulation 2015;132:1303-4. 10.1161/CIRCULATIONAHA.115.018750 pmid:26330415. 26330415

[19] Mozaffarian D, Benjamin EJ, Go AS, et al. American Heart Association Statistics Committee and Stroke Statistics Subcommittee. Heart disease and stroke statistics--2015 update: a report from the American Heart Association. Circulation 2015;131:e29-322. 10.1161/CIR.0000000000000152 pmid:25520374. 25520374

[20] Collins FS, Varmus H. A new initiative on precision medicine. N Engl J Med 2015;372:793-5. 10.1056/NEJMp1500523 pmid:25635347. 25635347 PMC5101938

[21] George J, Rapsomaniki E, Pujades-Rodriguez M, et al. How Does Cardiovascular Disease First Present in Women and Men? Incidence of 12 Cardiovascular Diseases in a Contemporary Cohort of 1,937,360 People. Circulation 2015;132:1320-8. 10.1161/CIRCULATIONAHA.114.013797 pmid:26330414. 26330414 PMC4590518

[22] Pujades-Rodriguez M, Timmis A, Stogiannis D, et al. Socioeconomic deprivation and the incidence of 12 cardiovascular diseases in 1.9 million women and men: implications for risk prediction and prevention. PLoS One 2014;9:e104671 10.1371/journal.pone.0104671 pmid:25144739. 25144739 PMC4140710

[23] Pujades-Rodriguez M, George J, Shah AD, et al. Heterogeneous associations between smoking and a wide range of initial presentations of cardiovascular disease in 1937360 people in England: lifetime risks and implications for risk prediction. Int J Epidemiol 2015;44:129-41. 10.1093/ije/dyu218 pmid:25416721. 25416721 PMC4339760

[24] Rapsomaniki E, Timmis A, George J, et al. Blood pressure and incidence of twelve cardiovascular diseases: lifetime risks, healthy life-years lost, and age-specific associations in 1·25 million people. Lancet 2014;383:1899-911. 10.1016/S0140-6736(14)60685-1 pmid:24881994. 24881994 PMC4042017

[25] Pujades-Rodriguez M, Duyx B, Thomas SL, Stogiannis D, Smeeth L, Hemingway H. Associations between polymyalgia rheumatica and giant cell arteritis and 12 cardiovascular diseases. Heart 2016;102:383-9. 10.1136/heartjnl-2015-308514 pmid:26786818. 26786818 PMC4789702

[26] Shah AD, Langenberg C, Rapsomaniki E, et al. Type 2 diabetes and incidence of cardiovascular diseases: a cohort study in 1·9 million people. Lancet Diabetes Endocrinol 2015;3:105-13. 10.1016/S2213-8587(14)70219-0 pmid:25466521. 25466521 PMC4303913

[27] Daskalopoulou M, George J, Walters K, et al. Depression as a Risk Factor for the Initial Presentation of Twelve Cardiac, Cerebrovascular, and Peripheral Arterial Diseases: Data Linkage Study of 1.9 Million Women and Men. PLoS One 2016;11:e0153838 10.1371/journal.pone.0153838 pmid:27105076. 27105076 PMC4841529

[28] Pujades-Rodriguez M, Duyx B, Thomas SL, et al. Rheumatoid Arthritis and Incidence of Twelve Initial Presentations of Cardiovascular Disease: A Population Record-Linkage Cohort Study in England. PLoS One 2016;11:e0151245 10.1371/journal.pone.0151245 pmid:26978266. 26978266 PMC4792375

[29] Denaxas SC, George J, Herrett E, et al. Data resource profile: cardiovascular disease research using linked bespoke studies and electronic health records (CALIBER). Int J Epidemiol 2012;41:1625-38. 10.1093/ije/dys188 pmid:23220717. 23220717 PMC3535749

[30] Gallagher AM, Puri S, van Staa TP. Linkage of the General Practice Research Database (GPRD) with other data sources. Pharmacoepidemiol Drug Saf 2011;20:S230-1.

[31] Mathur R, Bhaskaran K, Chaturvedi N, et al. Completeness and usability of ethnicity data in UK-based primary care and hospital databases. J Public Health (Oxf) 2014;36:684-92. 10.1093/pubmed/fdt116 pmid:24323951. 24323951 PMC4245896

[32] Herrett E, Gallagher AM, Bhaskaran K, et al. Data Resource Profile: Clinical Practice Research Datalink (CPRD). Int J Epidemiol 2015;44:827-36. 10.1093/ije/dyv098 pmid:26050254. 26050254 PMC4521131

[33] Herrett E, Shah AD, Boggon R, et al. Completeness and diagnostic validity of recording acute myocardial infarction events in primary care, hospital care, disease registry, and national mortality records: cohort study. BMJ 2013;346:f2350 10.1136/bmj.f2350 pmid:23692896. 23692896 PMC3898411

[34] Herrett E, Smeeth L, Walker L, Weston C. MINAP Academic Group. The Myocardial Ischaemia National Audit Project (MINAP). Heart 2010;96:1264-7. 10.1136/hrt.2009.192328 pmid:20659944. 20659944 PMC3505836

[35] Chisholm J. The Read clinical classification. BMJ 1990;300:1092 10.1136/bmj.300.6732.1092 pmid:2344534. 2344534 PMC1662793

[36] National Health Service. OPCS-4 Classification - NHS Connecting for Health.National Health Service, 2013.

[37] Department of Health. Sensible drinking: Report of an inter-departmental working group. Department of Health; 1995 http://tinyurl.com/ca3m5w3

[38] Noble M, McLennan D, Wilkinson K, et al. The English indices of deprivation 2007.Communities and Local Government, 2007.

[39] Sterne JAC, White IR, Carlin JB, et al. Multiple imputation for missing data in epidemiological and clinical research: potential and pitfalls. BMJ 2009;338:b2393 10.1136/bmj.b2393 pmid:19564179. 19564179 PMC2714692

[40] Rehm J, Irving H, Ye Y, Kerr WC, Bond J, Greenfield TK. Are lifetime abstainers the best control group in alcohol epidemiology? On the stability and validity of reported lifetime abstention. Am J Epidemiol 2008;168:866-71. 10.1093/aje/kwn093 pmid:18701442. 18701442 PMC2565735

[41] Bell S, Kivimäki M, Batty GD. Subgroup analysis as a source of spurious findings: an illustration using new data on alcohol intake and coronary heart disease. Addiction 2015;110:183-4. 10.1111/add.12708 pmid:25515832. 25515832 PMC4273867

[42] Bergmann MM, Rehm J, Klipstein-Grobusch K, et al. The association of pattern of lifetime alcohol use and cause of death in the European prospective investigation into cancer and nutrition (EPIC) study. Int J Epidemiol 2013;42:1772-90. 10.1093/ije/dyt154 pmid:24415611. 24415611 PMC3887563

[43] Mukamal KJ, Conigrave KM, Mittleman MA, et al. Roles of drinking pattern and type of alcohol consumed in coronary heart disease in men. N Engl J Med 2003;348:109-18. 10.1056/NEJMoa022095 pmid:12519921. 12519921

[44] Reynolds K, Lewis B, Nolen JDL, Kinney GL, Sathya B, He J. Alcohol consumption and risk of stroke: a meta-analysis. JAMA 2003;289:579-88. 10.1001/jama.289.5.579 pmid:12578491. 12578491

[45] Chiuve SE, Rimm EB, Mukamal KJ, et al. Light-to-moderate alcohol consumption and risk of sudden cardiac death in women. Heart Rhythm 2010;7:1374-80. 10.1016/j.hrthm.2010.05.035 pmid:20638933. 20638933 PMC2946479

[46] Albert CM, Manson JE, Cook NR, Ajani UA, Gaziano JM, Hennekens CH. Moderate alcohol consumption and the risk of sudden cardiac death among US male physicians. Circulation 1999;100:944-50. 10.1161/01.CIR.100.9.944 pmid:10468525. 10468525

[47] Larsson SC, Orsini N, Wolk A. Alcohol consumption and risk of heart failure: a dose-response meta-analysis of prospective studies. Eur J Heart Fail 2015;17:367-73. 10.1002/ejhf.228 pmid:25598021. 25598021

[48] Camargo CA Jr, , Stampfer MJ, Glynn RJ, et al. Prospective study of moderate alcohol consumption and risk of peripheral arterial disease in US male physicians. Circulation 1997;95:577-80. 10.1161/01.CIR.95.3.577 pmid:9024142. 9024142

[49] Stackelberg O, Björck M, Larsson SC, Orsini N, Wolk A. Alcohol consumption, specific alcoholic beverages, and abdominal aortic aneurysm. Circulation 2014;130:646-52. 10.1161/CIRCULATIONAHA.113.008279 pmid:24965567. 24965567

[50] Andreasen TH, Bartek J Jr, , Andresen M, Springborg JB, Romner B. Modifiable risk factors for aneurysmal subarachnoid hemorrhage. Stroke 2013;44:3607-12. 10.1161/STROKEAHA.113.001575 pmid:24193807. 24193807

[51] O’Donnell MJ, Xavier D, Liu L, et al. INTERSTROKE investigators. Risk factors for ischaemic and intracerebral haemorrhagic stroke in 22 countries (the INTERSTROKE study): a case-control study. Lancet 2010;376:112-23. 10.1016/S0140-6736(10)60834-3 pmid:20561675. 20561675

[52] Larsson SC, Wallin A, Wolk A, Markus HS. Differing association of alcohol consumption with different stroke types: a systematic review and meta-analysis. BMC Med 2016;14:178 10.1186/s12916-016-0721-4 pmid:27881167. 27881167 PMC5121939

[53] Romelsjö A, Allebeck P, Andréasson S, Leifman A. Alcohol, mortality and cardiovascular events in a 35 year follow-up of a nationwide representative cohort of 50,000 Swedish conscripts up to age 55. Alcohol Alcohol 2012;47:322-7. 10.1093/alcalc/ags021 pmid:22387338. 22387338

[54] Weikert C, Berger K, Heidemann C, et al. Joint effects of risk factors for stroke and transient ischemic attack in a German population: the EPIC Potsdam Study. J Neurol 2007;254:315-21. 10.1007/s00415-006-0358-x pmid:17345050. 17345050

[55] Camargo CA Jr, , Stampfer MJ, Glynn RJ, et al. Moderate alcohol consumption and risk for angina pectoris or myocardial infarction in U.S. male physicians. Ann Intern Med 1997;126:372-5. 10.7326/0003-4819-126-5-199703010-00005 pmid:9054281. 9054281

[56] Merry AH, Boer JM, Schouten LJ, et al. Smoking, alcohol consumption, physical activity, and family history and the risks of acute myocardial infarction and unstable angina pectoris: a prospective cohort study. BMC Cardiovasc Disord 2011;11:13 10.1186/1471-2261-11-13 pmid:21435252. 21435252 PMC3073941

[57] Zheng Y-L, Lian F, Shi Q, et al. Alcohol intake and associated risk of major cardiovascular outcomes in women compared with men: a systematic review and meta-analysis of prospective observational studies. BMC Public Health 2015;15:773 10.1186/s12889-015-2081-y pmid:26264040. 26264040 PMC4533962

[58] Bellis MA, Hughes K, Jones L, et al. Holidays, celebrations, and commiserations: measuring drinking during feasting and fasting to improve national and individual estimates of alcohol consumption. BMC Med 2015;13:113 10.1186/s12916-015-0337-0 pmid:25998218. 25998218 PMC4494693

[59] Arber S, McKinlay J, Adams A, Marceau L, Link C, O’Donnell A. Influence of patient characteristics on doctors’ questioning and lifestyle advice for coronary heart disease: a UK/US video experiment. Br J Gen Pract 2004;54:673-8.pmid:15353053. 15353053 PMC1326068

[60] Ferrari P, Licaj I, Muller DC, et al. Lifetime alcohol use and overall and cause-specific mortality in the European Prospective Investigation into Cancer and nutrition (EPIC) study. BMJ Open 2014;4:e005245 10.1136/bmjopen-2014-005245 pmid:24993766. PMC4091394 24993766

[61] Smyth A, Teo KK, Rangarajan S, et al. PURE Investigators. Alcohol consumption and cardiovascular disease, cancer, injury, admission to hospital, and mortality: a prospective cohort study. Lancet 2015;386:1945-54. 10.1016/S0140-6736(15)00235-4 pmid:26386538. 26386538

[62] Mortensen EL, Jensen HH, Sanders SA, Reinisch JM. Better psychological functioning and higher social status may largely explain the apparent health benefits of wine: a study of wine and beer drinking in young Danish adults. Arch Intern Med 2001;161:1844-8. 10.1001/archinte.161.15.1844 pmid:11493125. 11493125

[63] Ruidavets J-B, Ducimetière P, Evans A, et al. Patterns of alcohol consumption and ischaemic heart disease in culturally divergent countries: the Prospective Epidemiological Study of Myocardial Infarction (PRIME). BMJ 2010;341:c6077 10.1136/bmj.c6077 pmid:21098615. 21098615 PMC2990863

[64] Britton A, Ben-Shlomo Y, Benzeval M, Kuh D, Bell S. Life course trajectories of alcohol consumption in the United Kingdom using longitudinal data from nine cohort studies. BMC Med 2015;13:47 10.1186/s12916-015-0273-z pmid:25858476. 25858476 PMC4351673

[65] Dam MK, Hvidtfeldt UA, Tjønneland A, Overvad K, Grønbæk M, Tolstrup JS. Five year change in alcohol intake and risk of breast cancer and coronary heart disease among postmenopausal women: prospective cohort study. BMJ 2016;353:i2314 10.1136/bmj.i2314 pmid:27169583. 27169583 PMC5068920

[66] Bell S, Mehta G, Moore K, Britton A. Ten-year alcohol consumption typologies and trajectories of C-reactive protein, interleukin-6 and interleukin-1 receptor antagonist over the following 12 years: a prospective cohort study. J Intern Med 2017;281:75-85. 10.1111/joim.12544 pmid:27485145. 27485145 PMC5173424

[67] Britton A, Hardy R, Kuh D, Deanfield J, Charakida M, Bell S. Twenty-year trajectories of alcohol consumption during midlife and atherosclerotic thickening in early old age: findings from two British population cohort studies. BMC Med 2016;14:111 10.1186/s12916-016-0656-9 pmid:27473049. 27473049 PMC4967336

[68] Mukamal KJ, Ascherio A, Mittleman MA, et al. Alcohol and risk for ischemic stroke in men: the role of drinking patterns and usual beverage. Ann Intern Med 2005;142:11-9. 10.7326/0003-4819-142-1-200501040-00007 pmid:15630105. 15630105

[69] Holmes MV, Dale CE, Zuccolo L, et al. InterAct Consortium. Association between alcohol and cardiovascular disease: Mendelian randomisation analysis based on individual participant data. BMJ 2014;349:g4164 10.1136/bmj.g4164 pmid:25011450. 25011450 PMC4091648

[70] Glymour MM. Alcohol and cardiovascular disease. BMJ 2014;349:g4334 10.1136/bmj.g4334 pmid:25011451. 25011451

[71] Rehm J, Shield KD, Roerecke M, Gmel G. Modelling the impact of alcohol consumption on cardiovascular disease mortality for comparative risk assessments: an overview. BMC Public Health 2016;16:363 10.1186/s12889-016-3026-9 pmid:27121289. 27121289 PMC4848866

[72] Silverwood RJ, Holmes MV, Dale CE, et al. Alcohol-ADH1B Consortium. Testing for non-linear causal effects using a binary genotype in a Mendelian randomization study: application to alcohol and cardiovascular traits. Int J Epidemiol 2014;43:1781-90. 10.1093/ije/dyu187 pmid:25192829. 25192829 PMC4276061

[73] Orrow G, Kinmonth A-L, Sanderson S, Sutton S. Effectiveness of physical activity promotion based in primary care: systematic review and meta-analysis of randomised controlled trials. BMJ 2012;344:e1389 10.1136/bmj.e1389 pmid:22451477. 22451477 PMC3312793

[74] Kyu HH, Bachman VF, Alexander LT, et al. Physical activity and risk of breast cancer, colon cancer, diabetes, ischemic heart disease, and ischemic stroke events: systematic review and dose-response meta-analysis for the Global Burden of Disease Study 2013. BMJ 2016;354:i3857 10.1136/bmj.i3857 pmid:27510511. 27510511 PMC4979358

[75] Mons U, Müezzinler A, Gellert C, et al. CHANCES Consortium. Impact of smoking and smoking cessation on cardiovascular events and mortality among older adults: meta-analysis of individual participant data from prospective cohort studies of the CHANCES consortium. BMJ 2015;350:h1551 10.1136/bmj.h1551 pmid:25896935. 25896935 PMC4413837

[76] Department of Health. UK Chief Medical Officers’ alcohol guidelines review: Summary of the proposed new guidelines. Stationery Office; 2016. https://www.gov.uk/government/uploads/system/uploads/attachment_data/file/489795/summary.pdf

[77] National Health and Medical Research Council. Australian Guidelines to Reduce Health Risks from Drinking Alcohol. Australian Government; 2009:1-179. https://www.nhmrc.gov.au/_files_nhmrc/publications/attachments/ds10-alcohol.pdf

[78] American Heart Association. Alcohol and Heart Health. 2015. http://www.heart.org/HEARTORG/HealthyLiving/HealthyEating/Nutrition/Alcohol-and-Heart-Health_UCM_305173_Article.jsp

