# Supplementary material for: Association between clinically recorded alcohol consumption and initial presentation of 12 cardiovascular diseases: population based cohort study using linked health records
Source: BMJ. 2017 Aug 2;356:j909. doi: 10.1136/bmj.j909 (PMC5594422; doi:10.1136/bmj.j909)
Supplement: Supplementary file 1 — Appendix 1: Appendix [file bels037096.ww1.pdf]

Appendix: Supplementary material [posted as supplied by author]: *Association between clinically recorded alcohol consumption and initial presentation of 12 cardiovascular diseases: population based cohort study using linked health records* (Bell *et al*. 2017, *BMJ*, DOI: 10.1136/bmj.j909)

Table of contents

OVERVIEW OF MAJOR STUDIES ON ALCOHOL CONSUMPTION AND CVD.................. 2

STUDY DESCRIPTION 9

ASSESSMENT OF ALCOHOL CONSUMPTION IN CALIBER 10

OVERVIEW OF CODES/DATA SOURCES USED TO DEFINE EACH CARDIOVASCULAR ENDPOINT 15

MULTIPLE IMPUTATION.. 22

DISTRIBUTION OF INITIAL PRESENTATIONS BY DRINKING CATEGORY.................. 23

HAZARD RATIOS FOR NON-CVD MORTALITY AND, CHD AND STROKE, NOT OTHERWISE SPECIFIED 24

HAZARD RATIOS FOR MYOCARDIAL INFARCTION SUBTYPES.................................... 25

TESTS OF HETEROGENEITY ACROSS CVD ENDPOINTS BY DRINKING CATEGORY 26

SUPPLEMENTARY ANALYSES 27

ADJUSTED FOR AGE AND SEX ONLY 28
ADDITIONAL ADJUSTMENT FOR SYSTOLIC BLOOD PRESSURE, DIABETES STATUS, BODY MASS INDEX, HDL-CHOLESTEROL, USE OF STATINS OR BLOOD PRESSURE LOWERING MEDICATION, AND WHETHER OFFERED DIETARY ADVICE 29
ANALYSES BY GENDER 30
P-VALUES FOR INTERACTIONS BY SEX 30
MAIN ANALYSES LIMITED TO MEN.. 31
MAIN ANALYSES LIMITED TO WOMEN 32
USING ONLY SECONDARY CARE AND MORTALITY BASED ENDPOINTS 33
FATAL ENDPOINTS ONLY.. 34
ANALYSES USING DATA COLLECTED POST-2004 35
COMPLETE CASE ANALYSIS 36
ANALYSIS RESTRICTED TO NEVER SMOKERS 37
ANALYSIS RESTRICTED TO SMOKERS.. 38
ANALYSIS RESTRICTED TO THOSE WITH BMI VALUES IN THE NORMAL RANGE............................ 39
ANALYSIS RESTRICTED TO THOSE WITH BMI VALUES CONSIDERED OVERWEIGHT 40
ANALYSIS RESTRICTED TO THOSE WITH BMI VALUES CONSIDERED OBESE.................................. 41

SUPPLEMENTARY REFERENCES.. 42

Overview of major studies on alcohol consumption and CVD

There are multiple systematic reviews and meta-analyses of the association between alcohol consumption and aggregated CVD[1–7] as well as CVD biomarkers.[4,8,9] Most have shown that moderate levels of alcohol intake are associated with a lower risk of CVD morbidity and mortality, as well as more favourable cardiovascular health profiles in general, than non-drinkers. However, there is a growing scepticism around this observation, with a series of recent commentary pieces pointing out a number of methodological shortcomings in the evidence that the U-shape is based on.[10–12] These include failure to have decomposed the current non-drinking group into life-long abstainers, former drinkers and those who drink on an occasional (but not weekly basis). It is known that former drinkers have an increased risk of CVD mortality[13] compared to life-long non-drinkers; therefore combining these two groups is likely to lead to the protective effects of moderate drinking being over estimated in being compared to a non-drinking group that consists of former drinkers, who may have quit for health reasons. Similarly, it has been shown that the onset of ill health is associated with a reduction in regular alcohol consumption to drinking on an occasional basis,[14] therefore combining these individuals with non-drinkers also introduces bias.

Evidence from short-term alcohol feeding interventions has shown that moderate drinking is related to higher levels of high-density lipoprotein cholesterol (HDL-C) and adiponectin, as well as lower levels of fibrinogen, for which it has been argued reflects indirect evidence of a causal association of moderate alcohol conferring a lower risk of experiencing CVD outcomes.[8] However, findings from large scale Mendelian randomisation studies as well as pharmacological trials suggest that the causal role of these biomarkers in the development of CVD is at best unclear.[15–19] Furthermore, a recent individual participant data Mendelian randomisation analysis of the association alcohol consumption (using the rs1229984 variant in the alcohol dehydrogenase 1B gene (*ADH1B*) as an instrumental variable) found that individuals with a genetic variant which predisposes them to low-level alcohol intake or non-drinking actually had a lower risk of CHD and a generally better cardiovascular health profile than those who did not carry the genetic variant.[20] This is counter to the association typically found in observational studies. However, it should be noted that in a related paper from the same consortium, that non-linear associations were found for alcohol intake and a range of biomarkers associated (but not necessarily causally related) with CVD including non-HDL cholesterol, BMI, waist circumference and C-reactive protein, but not for HDL-C, triglycerides or interleukin-6.[21]

Given differences in the association of alcohol consumption and different CVD biomarkers, it could be hypothesised that moderate alcohol consumption may be protective for some CVDs but not others.[22] However, the evidence base for specific CVD phenotypes is sparse in comparison to that of aggregated CVD outcomes – with the majority of research focussing on acute myocardial infarction or stroke (total and broad categories of ischaemic or haemorrhagic). There have been calls for further research into the association of alcohol consumption and deeper phenotypes of CVD[23] to further our understanding of the role of alcohol consumption in the development or prevention of individual CVDs in order to improve risk prediction at both an individual and population level. However, few studies are sufficiently powered to examine individual CVDs and fewer still are in a position whereby they are also able to disaggregate the current non-drinker group into non-drinkers, former drinkers and occasional drinkers. We provide an overview of research from major investigator led prospective observational studies as well as meta-analyses of the topic of alcohol consumption and specific CVDs in Table A. As noted above, there are a number of existing studies for primary endpoints such as myocardial infarction and ischaemic stroke, with meta-analyses and Mendelian randomisation studies also available for these topics. However, there is a substantially smaller evidence base for other outcomes such as heart failure, cardiac arrest/sudden coronary death, angina, transient ischaemic

attack, peripheral arterial disease, abdominal aortic aneurysm, unheralded CHD death and haemorrhagic stroke subtypes (subarachnoid or intracerebral haemorrhage), and many of the investigator led cohort studies have in some manner combined different current non-drinking groups into an aggregate category.[24]

Table A - Overview of major studies of alcohol consumption and individual CVDs

Outcome	Major[a] investigator led (IL) prospective observational studies / meta-analysis	Disaggregated non-drinking group in major IL study?	Initial presentation[b]	Electronic health records study	Evidence before this study	What this study adds
**Stable angina**	Yes[25]	Former drinkers not separated from non-drinking group.	No	No	In a single study of US Male Physicians moderate alcohol consumption has been shown to be associated with a lower risk of developing stable angina.	We show that the protective effect of moderate drinking holds even after decomposing the current non-drinker group into non-drinkers, former drinkers and occasional drinkers. We also observed evidence of a protective effect of heavy drinking for risk of initially presenting with stable angina - albeit non-significant.
**Unstable angina**	Yes[26]	Yes	No	No	Regular moderate alcohol consumption associated with a lower risk of UA.	We found that non-drinkers and former drinkers had greater risk of developing unstable angina compared to moderate drinkers. We observed little difference in risk across current drinkers whether it be occasional or heavy drinking.
**Myocardial infarction**	Yes[25–32]	Varies between studies; most have combined non-drinkers, former drinkers and	No	No	Most observational studies show a protective effect of alcohol consumption – even at high levels compared to non-drinking. Mendelian randomisation studies are mixed.	Our findings are concordant with existing epidemiological studies. We show that the protective effect of increasing alcohol consumption exists even when focussing only on initial presentation in the

		occasional drinkers in some way.				absence of intercurrent other cardiovascular disease. We are also the first to examine the association between alcohol consumption and MI subtypes, including STEMI, nSTEMI and MI, NOS – finding no heterogeneity across endpoints.
**Unheralded CHD death**	No	--	No	No	No studies specifically on unheralded CHD death.	We present the first findings of the association between alcohol consumption and unheralded CHD death, showing that non-drinkers are much more likely to more likely to present with coronary death with no prior symptomatic presentations than moderate drinkers. Similarly we see large increases in risk amongst former drinkers consistent with the sick quitting hypothesis. We also observed increases in risk amongst occasional drinkers and heavy drinkers in comparison to moderate drinkers.
**Heart failure**	Yes[33–37]	Largest study (126 236 participants) split non-drinkers into never, ex- and occasional.	No	No	Recent meta-analysis contained eight prospective studies, with a total of 202 378 participants and 6211 cases of heart failure. A non-linear association was found such that moderate alcohol	Our findings are consistent with the most recent meta-analysis on the topic, seeing elevated risk of initially presenting with heart failure amongst non-, former and occasional drinkers. This holds in the present study that has

					consumption was protective compared to non-drinking.	more than double the number of heart failure events (14359) and excludes intercurrent presentations of CVD. We also demonstrate that heavy drinkers have an increased risk of heart failure.
**Cardiac arrest/SCD**	Yes[38,39]	In men; no non-drinking group (mixed with occasional drinkers). In women, former drinkers separated from never drinkers; occasional drinkers mixed with referent category.	No	No	Protective effects observed for moderate consumption compared to non-drinking observed in US Male Physicians and Nurses. In men, risk decreases with increasing alcohol intake whilst in women the association was non-linear.	In pooled analyses the association between each non-drinking category and risk of initially presenting with cardiac arrest/SCD compared to moderate drinking was non-significant. In in contrast to previous studies in men, we did not find a linear dose-response association between alcohol intake and lower risk of SCD, in fact, we observed the opposite.
**Transient ischaemic attack**	Yes[40]	No; never, ex- and occasional drinkers mixed with moderate drinkers	No	No	Little research on alcohol consumption and TIA. Single report found that heavy drinkers had an increased risk of experiencing a TIA compared to non-heavy drinkers.	We are the first to report findings for the association of non-drinking and TIA, finding no protective effects of moderate drinking in comparison to non-drinking. We found a marginally significant increased risk of presenting with TIA amongst heavy drinkers.
**Ischaemic stroke**	Yes[3,41–53]	Varies between studies; many	No	No	Most recent meta-analysis consisted of 27 studies reporting data on 1 425 513	In a sample larger than the most recent meta-analysis we show that non-drinkers have a

		have combined non-drinkers, former drinkers and occasional drinkers in some way.			individuals. Found moderate drinking was associated with a lower risk of ischaemic stroke compared to non-drinking (no significant difference observed for heavy drinking and ischaemic stroke). *ADH1C* $\gamma 1/\gamma 2$ polymorphism not associated with risk of ischaemic stroke. Carriers of *ADH1B* A-allele have lower odds of ischaemic stroke.	higher risk of ischaemic stroke compared to moderate drinkers. We show a heightened risk of stroke in former drinkers in comparison to moderate drinkers and a marginally significantly elevated risk of ischaemic stroke amongst heavy drinkers.
**Subarachnoid haemorrhage**	Yes[42,54–57]	Former drinkers combined with never drinkers	No	No	Evidence of a dose-response association between amount of alcohol consumed and risk of subarachnoid haemorrhage in the most recent meta-analysis. No accounting for former drinkers.	In contrast to previous smaller studies, we observed no difference in risk of initially presenting with a subarachnoid haemorrhage in any drinking category as compared to moderate drinking.
**Intracerebral haemorrhage**	Yes[42,57,58]	Former drinkers combined with never drinkers	No	No	Meta-analysis revealed increased risk of ICH with increasing alcohol intake. Large scale study of subtype of intraparenchymal haemorrhage showed non-drinkers as well as regular drinkers had an increased risk of IHS compared to occasional drinkers.	We found that heavy drinkers have a much larger risk of initially presenting with Intracerebral haemorrhage than moderate drinkers after excluding incurrent cardiovascular conditions.
**Peripheral arterial disease**	Yes[59]	Never, former and occasional drinkers were combined.	No	No	In US male physicians, increasing alcohol consumption was associated with a lower risk of PAD; however, this was compared to a mixed reference group and	We observed elevated risk of initially presenting with PAD amongst all types of non-drinker in comparison to moderate drinkers. We are also the first the report the

					did not specifically separate heavy drinkers from those who drink in moderation.	association between heavy drinking and PAD, finding that heavy drinkers are more likely to initially present with PAD than their moderate drinking counterparts.
**Abdominal aortic aneurysm**	Yes[60–62]	In largest and most recent study, never and former drinkers separated but occasional drinkers combined with moderate.	No	No	Moderate alcohol consumption was associated with a lower hazard of AAA. The association between higher doses of alcohol (> 120g and 60g per week for men and women, respectively) and risk of AAA remain unknown.	We are the first to report on the association between heavy drinking and AAA, finding no significant difference in risk of initial presentation between moderate and heavy drinkers.

[a] Major defined as a prospective observational study with a sample size ≥ 10,000 participants and validated clinical events. [b] Defined as assessing the first presentation in the absence of intercurrent other cardiovascular disease.

SCD; sudden coronary death.

Study description

We included 1,937,360 anonymised patients from the CALIBER (CArdiovascular research using LInked Bespoke studies and Electronic health Records) programme.[63] The cohort used in the present study was drawn from patients registered with Clinical Practice Research Datalink (CPRD)[64] general practices in England that consented to data linkage (approximately 5% of the UK population). We used an open cohort design, where participants joined the cohort when they met the inclusion criteria at any point between 1st January 1997 and 25th March 2010 (the last CPRD data submission). Patients were included in cohort if they were aged ≥ 30 years, had at least one year of electronic health record data which met CPRD data quality standards, and had no record indicating any cardiovascular disease prior to study entry. Patients for who sex was not recorded and those pregnant within six months of the eligibility date were also excluded. Patients were followed up until the date of an initial presentation of one of our cardiovascular endpoints or were censored on the date of leaving the practice/last data submission from their practice. Patients who died before 1st January 2001 were excluded as cause-specific mortality data was not available for them (see study flow diagram; Figure A). Patient CPRD data were further linked with three other data sources; the Myocardial Ischaemia National Audit Project registry (MINAP);[65] Hospital Episodes Statistics (HES); and the Office for National Statistics (ONS). CPRD provides primary care data on health behaviours, diagnoses, investigations, procedures and prescriptions; and its accuracy and completeness are regularly audited. MINAP is a national registry of patients hospitalised with acute coronary syndromes in England and Wales. HES provides information on all hospital admissions and ONS cause-specific mortality records for all deaths in England and Wales. Information is coded using the hierarchical clinical coding schemes (Read[66], the International Statistical Classification of Diseases and Health Related Problems, 10th revision[67], and Office of the Population Censuses and Surveys Classification of Interventions and Procedures[68]).

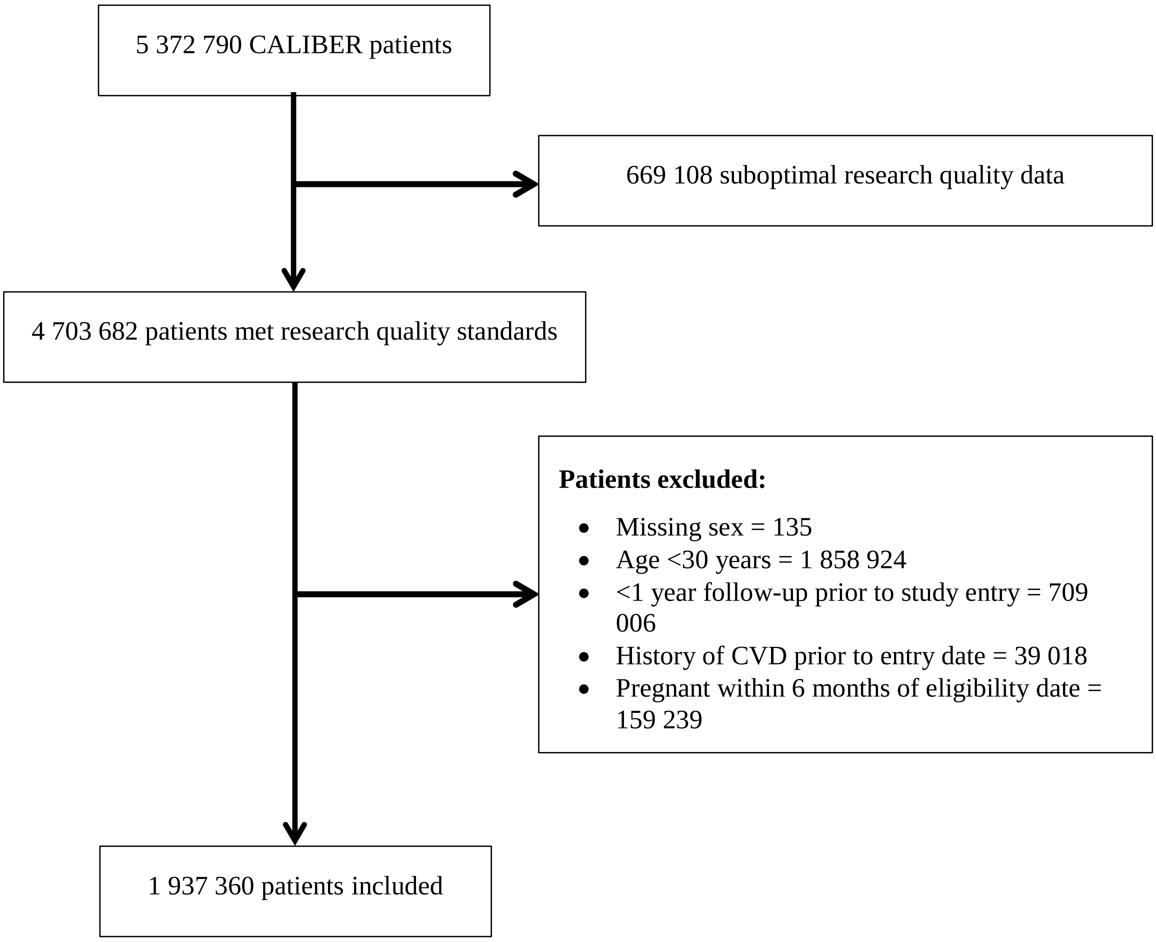

Figure A - Study flow diagram

Assessment of alcohol consumption in CALIBER

Self-reported alcohol consumption was collected prospectively and coded by general practitioners or practice nurses on the consultation date in CPRD. The most recent alcohol consumption record in the five years before entry into the study was used to classify participants drinking behaviour. In light of current debates on the U/J-shaped relationship observed between alcohol consumption and aggregated CVD outcomes[10] five drinking categories were defined including: (1) non-drinkers (Read[66] codes such as "tee-total" and "non-drinkers"), former drinkers (those with codes for "stopped drinking alcohol" and/or "ex-drinker"), occasional drinkers (those with codes for "drinks rarely" and/or "drinks occasionally"), current moderate drinkers (those who had a code for current alcohol consumer and an indicator of whether they drank within daily [32g or 24g of alcohol for men and women respectively] and/or weekly [168g of alcohol for men and 112g for women] recommended sensible drinking limits for the UK at the time of observation[69]) and current heavy drinkers (defined as those who exceeded daily and/or weekly sensible drinking limits). We also utilised data fields with information entered on daily and/or weekly amount of alcohol consumed to define participants as non-drinkers, moderate drinkers (drank within daily and/or weekly guidelines) and heavy drinkers. Weekly alcohol data was available as a continuous variable, so we were able to classify consumption using standard thresholds (outlined above and in Figure B). Data on daily alcohol intake was entered using categories of: (1) < 1 UK unit (8 grams of ethanol), (2) 1-2 UK units, (3) 3-6 UK units, (4) 7-9 UK units, and (5) > 9 UK units [Read[66] codes 1362.00-1366.00], for which we defined moderate drinking as anything >1 UK unit but less than 3 (women) or 7 (men) UK units. Unfortunately information on binge drinking was only available for a select minority of the cohort (~100 people) therefore a separate category for this drinking behaviour was not defined (but these patients were coded as heavy drinkers). We reclassified non-drinkers as former drinkers if they had any record of drinking recorded in their entire clinical record entered on CPRD prior to study entry (in cases whereby non-drinkers had no record of drinking before entering the study we assumed that they were not former drinkers). This resulted in 19,853 (out of 184,747; 10.7%) non-drinkers being recoded as former drinkers, a further 6,826 (3.7%) participants were reclassified through having a positive history of alcohol abuse. A flow diagram outlining our coding algorithm is presented in Figure B and the exact Read codes used to define drinking categories are presented in Table B.

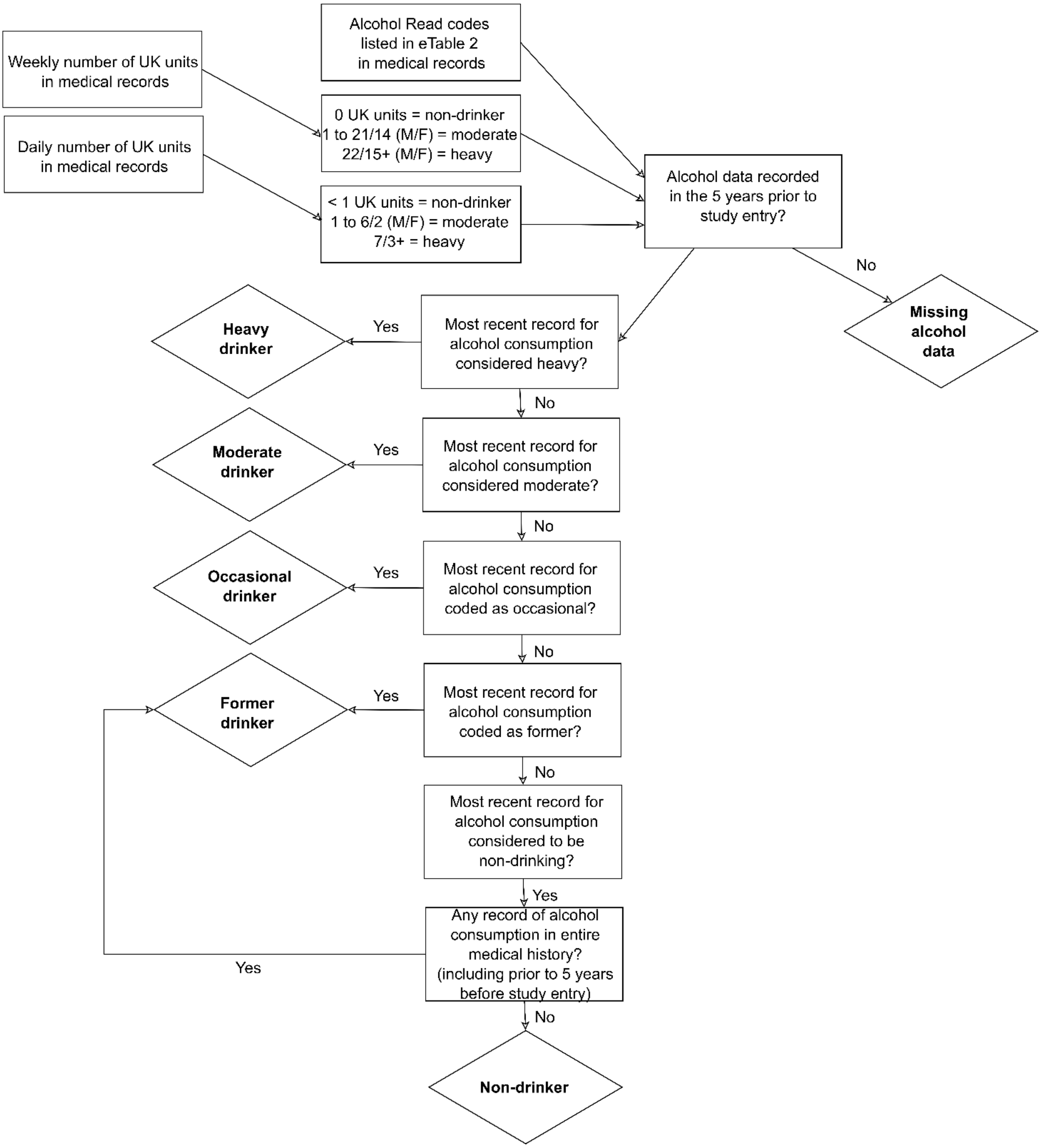

Figure B - Alcohol coding algorithm in CALIBER

Table B - Read codes used to construct the clinically recorded alcohol consumption variable

Read code	Read term	Drinking category
**1361.00**	Teetotaller	Non-drinker
**1361.11**	Non drinker alcohol	Non-drinker
**1361.12**	Non-drinker alcohol	Non-drinker
**136M.00**	Current non drinker	Non-drinker
**1367.00**	Stopped drinking alcohol	Former drinker
**136A.00**	Ex-trivial drinker (<1u/day)	Former drinker
**136B.00**	Ex-light drinker - (1-2u/day)	Former drinker
**136C.00**	Ex-moderate drinker - (3-6u/d)	Former drinker
**136D.00**	Ex-heavy drinker - (7-9u/day)	Former drinker
**136E.00**	Ex-very heavy drinker-(>9u/d)	Former drinker
**1362.11**	Drinks rarely	Occasional drinker
**1362.12**	Drinks occasionally	Occasional drinker
**136F.00**	Spirit drinker	Moderate drinker
**136G.00**	Beer drinker	Moderate drinker
**136H.00**	Drinks beer and spirits	Moderate drinker
**136I.00**	Drinks wine	Moderate drinker
**136J.00**	Social drinker	Moderate drinker
**136L.00**	Alcohol intake within recommended sensible limits	Moderate drinker
**136N.00**	Light drinker	Moderate drinker
**136O.00**	Moderate drinker	Moderate drinker
**1D19.00**	Pain in lymph nodes after alcohol consumption	Moderate drinker
**2577.00**	O/E - breath - alcohol smell	Moderate drinker
**2577.11**	O/E - alcoholic breath	Moderate drinker
**136K.00**	Alcohol intake above recommended sensible limits	Heavy drinker
**136P.00**	Heavy drinker	Heavy drinker
**136Q.00**	Very heavy drinker	Heavy drinker
**136S.00**	Hazardous alcohol use	Heavy drinker
**136T.00**	Harmful alcohol use	Heavy drinker
**136W.00**	Alcohol misuse	Heavy drinker
**13ZY.00**	Disqualified from driving due to excess alcohol	Heavy drinker
**E23..12**	Alcohol problem drinking	Heavy drinker
**E250.00**	Nondependent alcohol abuse	Heavy drinker
**E250000**	Nondependent alcohol abuse, unspecified	Heavy drinker
**E250100**	Nondependent alcohol abuse, continuous	Heavy drinker
**E250300**	Nondependent alcohol abuse in remission	Heavy drinker
**E250z00**	Nondependent alcohol abuse NOS	Heavy drinker
**ZV11311**	[V]Problems related to lifestyle alcohol use	Heavy drinker
**136R.00**	Binge drinker	Heavy drinker
**E250200**	Nondependent alcohol abuse, episodic	Heavy drinker
**E250.11**	Drunkenness NOS	Heavy drinker
**E250.12**	Hangover (alcohol)	Heavy drinker
**E250.13**	Inebriety NOS	Heavy drinker

**E250.14**	Intoxication - alcohol	Heavy drinker
**R103.00**	[D]Alcohol blood level excessive	Heavy drinker
**U81..00**	[X]Evidence of alcohol involvement determined by level of intoxication	Heavy drinker

We provide analyses of the association of these drinking categories with cardiovascular traits as means of validating the clinically recorded alcohol consumption measure we derived using CPRD data in Figure C (with adjustments for age, age-squared and sex). Consistent with observational studies we found that higher alcohol intake was associated with increased levels of HDL-C and total cholesterol as well as elevated blood pressure.[20] We also observed that heavy drinkers were more likely to be smokers. Diabetes was more prevalent in non, former and occasional drinkers at baseline, consistent with the sick-quitter[70] and sick-non-starter[71] hypotheses. Higher alcohol intake was also associated with lower odds of prevalent diabetes and HbA1c levels which is concordant with cross-sectional studies[72,73] Increased alcohol consumption was also associated with lower levels of creatinine,[74] but non- and former-drinkers did not have elevated levels compared to moderate drinkers. Non-drinkers, former drinkers and occasional drinkers all had higher BMI than moderate drinkers whereas heavy drinkers did not significantly differ from those who drank in moderation. Higher levels of alcohol intake were also associated with higher levels of gamma-glutamyl transferase.[75]

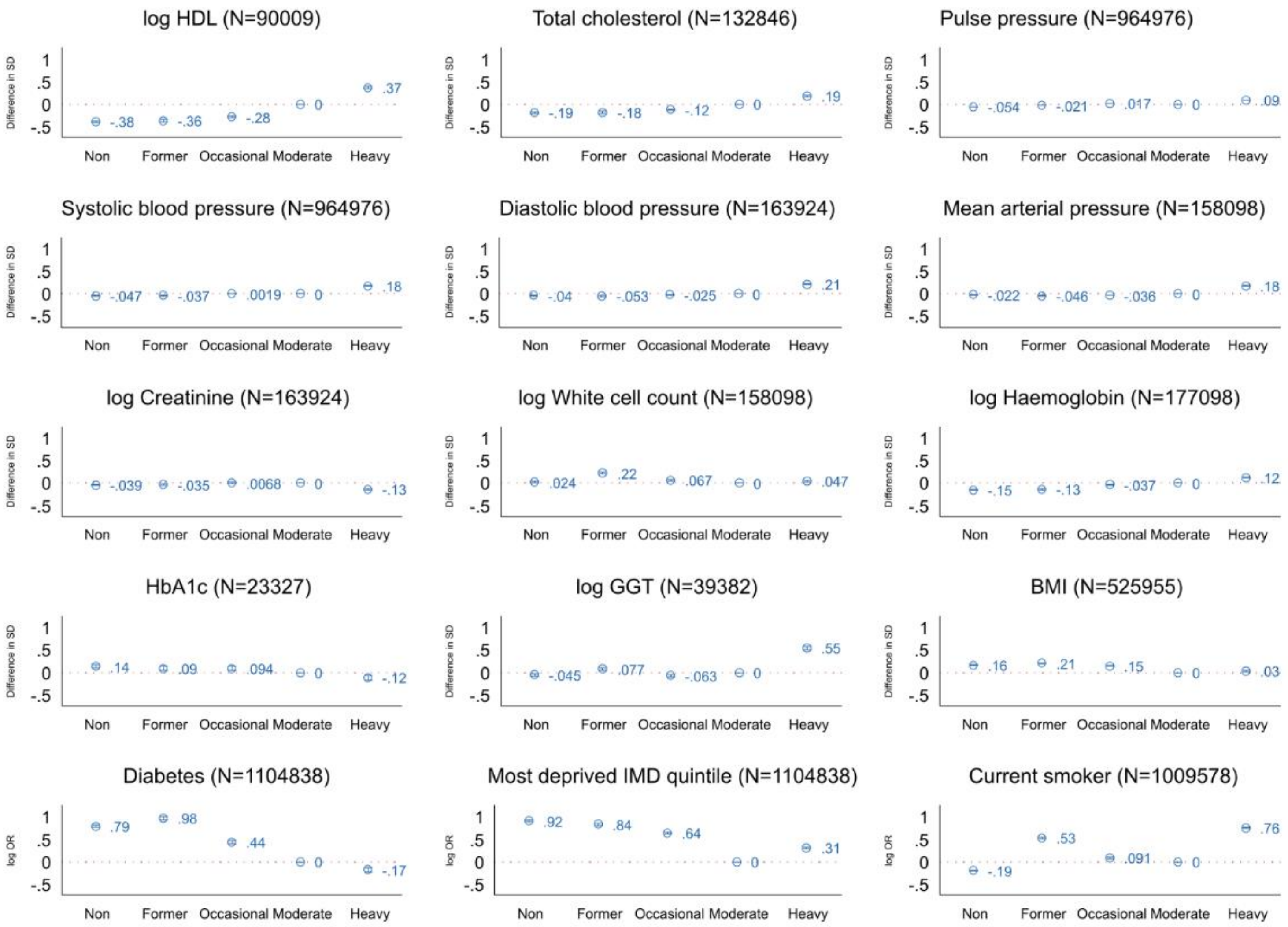

Figure C - Association of clinically recorded alcohol consumption categories and cardiovascular related traits

Overview of codes/data sources used to define each cardiovascular endpoint

As outlined in the main text we used multiple endpoints based on the first recorded diagnosis of the 12 most common symptomatic CVD manifestations, including: chronic stable angina (SA), unstable angina (UA), acute myocardial infarction (MI), unheralded coronary heart disease death (UCD), heart failure (HF), cardiac arrest/sudden cardiac death (SCD), transient ischaemic attack (TIA), ischaemic and haemorrhagic (subarachnoid and intracerebral haemorrhages) stroke, peripheral arterial disease (PAD), and abdominal aortic aneurysm (AAA). We were interested in only the first occurrence of any CVD, so subsequent events (e.g. ischaemic stroke occurring after TIA) were not analysed.

Diagnoses could occur in primary or secondary care, or at death, and were defined/validated using multiple sources; including a combination of symptoms, diagnoses (including the use of additional information from ECG findings and troponin values) and medication prescriptions. There is an extensive literature demonstrating that CPRD patients are representative of the UK population in terms of age, gender, ethnicity and overall mortality as well as the validity of risk factor and disease endpoints defined using multiple electronic health record (EHR) sources.[76–84] We have further confidence in the validity of linked EHR in having seen in this study, and others,[85–89] that associations were broadly similar when restricting analysis to data sources; each of which will have different health care practitioners who are responsible for entering diagnoses.

Details of the data sources and relevant codes used to define each endpoint are outlined in Table C.

Table C - Codes and data sources used for cardiovascular diseases in CALIBER

Endpoint	CPRD – Read codes	MINAP – specific disease registry	HES – OPCS 4 hospital procedures	HES – ICD 10 hospital diagnoses†	ONS – ICD 10 causes of death‡
**Stable angina**	G33..00: Stable Angina.<br>G33z.00: Angina pectoris NOS + 25 other codes for diagnosis of stable angina pectoris.<br>30 codes for evidence of coronary artery disease at angiography (CT,MR, invasive or not specified).<br>151 Read codes for evidence of myocardial ischaemia (Resting ECG, exercise ECG, stress echo, radioisotope scan).<br>Two or more successive prescriptions for anti-anginals.	N/U	K40-K46: Coronary artery bypass graft.<br>K49,K50 and K75: Percutaneous coronary intervention, not within 30 days of an acute coronary syndrome.	I20: Stable angina pectoris excluding unstable angina (I20.0).	N/U
**Unstable angina**	G311.13/G311100: Unstable angina.<br>G233200: Angina at rest.<br>G311400: Worsening angina + 13 other codes.	Discharge diagnosis of unstable angina, no raised ST elevation. No raised troponin levels.	N/U	I20.0: Unstable or worsening angina.<br>I24: Acute ischaemic heart disease.<br>I24.0: Coronary thrombosis not resulting in MI.<br>I24.8: Other forms of ischaemic heart	N/U

Endpoint	CPRD – Read codes	MINAP – specific disease registry	HES – OPCS 4 hospital procedures	HES – ICD 10 hospital diagnoses†	ONS – ICD 10 causes of death‡
				disease.<br>I24.9: Acute ischaemic heart disease, unspecified.	
**Coronary heart disease not otherwise specified**	G3…00: Ischaemic heart disease + 90 other codes including CHD NOS, chronic ischaemic heart disease, silent myocardial infarction.	N/U	N/U	CHD NOS, chronic ischaemic heart disease, silent MI (I25) excluding.I25.2, old MI.	N/U
**Acute Myocardial Infarction (MI)**	G30X000: Acute ST segment elevation myocardial infarction.<br>G307100: Acute non-ST segment elevation myocardial infarction.<br>G30..14: Heart attack.<br>G30..15: MI Acute myocardial infarction + 60 other codes as Acute MI not otherwise specified.	MI with or without ST elevation based on initial electrocardiogram findings, raised troponins and clinical diagnosis.	N/U	I21: Acute myocardial infarction.<br>I23: Current complications of acute MI.	N/U
**Unheralded coronary death**	Any CVD excluded.	Any CVD excluded.	Any CVD excluded.	Any CVD excluded.	I20: Angina Pectoris.<br>I21: Acute MI.<br>I22: Subsequent MI.<br>I23: Certain current complications following acute MI.<br>I24: Other acute ischaemic heart diseases.<br>I25: Chronic ischaemic

Endpoint	CPRD – Read codes	MINAP – specific disease registry	HES – OPCS 4 hospital procedures	HES – ICD 10 hospital diagnoses†	ONS – ICD 10 causes of death‡
					heart disease.
**Heart failure**	G58..00: Heart Failure + 92 other Read codes for heart failure diagnosis.	N/U	N/U	I50: Heart failure.<br>I11.0: Hypertensive heart disease with (congestive) heart failure.<br>I13.0: Hypertensive heart and renal disease with (congestive) heart failure.<br>I13.2: Hypertensive heart and renal disease with both (congestive) heart failure and renal disease.	I50 Heart failure.<br>I11.0 Hypertensive heart disease with (congestive) heart failure.<br>I13.0: Hypertensive heart and renal disease with (congestive) heart failure.<br>I13.2: Hypertensive heart and renal disease with both (congestive) heart failure and renal disease
**Ventricular arrhythmias, cardiac arrest and sudden cardiac death**	G574.00: Ventricular fibrillation and flutter.<br>G757.00: Cardiac arrest + 35 other Read codes for ventricular fibrillation, asystole, cardiac arrest, cardiac resuscitation, electro-mechanical dissociation.<br>G575100: Sudden cardiac death.	N/U	X50: Implanted cardiac defibrillation device.<br>K59: Implantation, revision and renewal of cardiac defibrillator.	I46: Cardiac arrest.<br>I47.0: Re-entry ventricular arrhythmia.<br>I47.2: Ventricular tachycardia.	I46: Cardiac arrest.<br>I47.0: Re-entry ventricular arrhythmia.<br>I47.2: Ventricular tachycardia
**Transient ischaemic attack**	Fyu5500: [X]Other transient cerebral ischaemic attacks + related symptoms + 5 other Read codes.	N/U	N/U	G458: Other transient cerebral ischaemic attacks and related syndromes.<br>G459: Transient cerebral ischaemic	N/U

Endpoint	CPRD – Read codes	MINAP – specific disease registry	HES – OPCS 4 hospital procedures	HES – ICD 10 hospital diagnoses†	ONS – ICD 10 causes of death‡
				attack, unspecified.	
**Ischaemic stroke**	G64..11: CVA – cerebral artery occlusion, G64..13 Stroke due to cerebral arterial occlusion. G6W..00: Cerebral infarction due to unspecified occlusion/stenosis of precerebral arteries. G6X..00: Cerebral infarction due to unspecified occlusion/stenosis of cerebral arteries plus 8 other codes.	N/U	Stroke NOS with carotid endarterectomy or stenting within 90 days (OPCS codes L294, L295, L311, L314; Read codes 7A20300 + 4 others).	I63: Cerebral infarction.	I63: Cerebral infarction.
**Subarachnoid haemorrhage**	G601.00:Subarachnoid haemorrhage from carotid siphon and bifurcation. G602.00: Subarachnoid haemorrhage from middle cerebral artery. G60X.00: Subarachnoid haemorrhage from intracranial artery, unspecified.	N/U	N/U	I60: Subarachnoid haemorrhage.	I60: Subarachnoid haemorrhage.

Endpoint	CPRD – Read codes	MINAP – specific disease registry	HES – OPCS 4 hospital procedures	HES – ICD 10 hospital diagnoses†	ONS – ICD 10 causes of death‡
**Intracerebral haemorrhage**	Gyu6F00: [x] Intracerebral haemorrhage in hemisphere, unspecified + 16 other codes.	N/U	N/U	I61: Intracerebral haemorrhage.	I61: Intracerebral haemorrhage.
**Stroke not otherwise specified**	G66..11: Cerebrovascular accident unspecified + 14 other Read codes.	N/U	U54.3: Delivery of rehabilitation for stroke.	I64: Stroke not specified as haemorrhage or infarction.<br>G463-G467: Stroke syndromes.	I64: Stroke not specified as haemorrhage or infarction.<br>I672: Cerebral atherosclerosis.<br>I679: Cerebrovascular disease, unspecified.
**Peripheral arterial disease**	63 codes for lower limb peripheral arterial disease diagnosis (including diabetic PAD, gangrene, arterial thrombosis of the leg and intermittent claudication).<br>Evidence of atherosclerosis of iliac and lower limb arteries based on angiography or Dopplers.	N/U	L50-L54: Bypass, reconstruction and other open operations on iliac artery.<br>L58-L60, L62: Bypass, reconstruction, transluminal operations or other open operations of femoral artery.<br>L65: Revision of reconstruction of artery.	I70.2: atherosclerosis of arteries of extremities.<br>I73.9: Peripheral vascular disease intermittent claudication<br>E10.05,E11-E14: Peripheral complications of diabetes including gangrene, insulin dependent diabetes mellitus, non-insulin-dependent diabetes mellitus, malnutrition-related diabetes mellitus, other specified diabetes	I70.2: Atherosclerosis of arteries of extremities.<br>I73.9: Peripheral vascular disease intermittent claudication.<br>Peripheral complications of diabetes including gangrene 0.5 suffix of E10: Insulin dependent diabetes mellitus, E11: Non-insulin-dependent diabetes mellitus, E12: Malnutrition-related diabetes mellitus, E13: Other specified diabetes mellitus, E14:

Endpoint	CPRD – Read codes	MINAP – specific disease registry	HES – OPCS 4 hospital procedures	HES – ICD 10 hospital diagnoses†	ONS – ICD 10 causes of death‡
				mellitus, unspecified diabetes mellitus.	Unspecified diabetes mellitus
**Abdominal aortic aneurysm**	G714.00: AAA without mention of rupture + 12 more codes for AAA diagnosis.<br>42 codes for AAA procedures.	N/U	L16: Extra anatomic bypass of aorta.<br>L18-L23: Replacement of aneurysmal segment of aorta, bypass of segment of aorta, plastic repair of aorta.<br>L25-L28: Transluminal or endovascular insertion of stent on aneurysmal segment of aorta	I71.3: Ruptured AAA.<br>171.4: AAA without rupture.<br>I71.5: Ruptured thoraco-abdominal aortic aneurysm.<br>I71.6: Thoracoabdominal aortic aneurysm without mention of rupture.<br>I71.8: Aortic aneurysm of unspecified site, ruptured.<br>I71.9: Aortic aneurysm of unspecified site, without mention of rupture.	I71.3: Ruptured AAA.<br>I71.4: AAA without rupture.<br>I71.5: Ruptured thoraco-abdominal aortic aneurysm.<br>I71.6: Thoracoabdominal aortic aneurysm without mention of rupture.<br>I71.8: Aortic aneurysm of unspecified site, ruptured.<br>I71.9: Aortic aneurysm of unspecified site, without mention of rupture.

Note: AAA, aortic abdominal aneurysm; CVD, cardiovascular disease; MI, myocardial infarction; NOS, not otherwise specified; N/U = not used in definition; OPCS, Office of Population Censuses and Surveys Classification of Interventions and Procedures. †Primary cause of admission. ‡Underlying cause of death.

Multiple imputation

Multiple imputation was carried out using the mi command in the statistical package Stata, to replace missing values in exposure and risk factor variables under a missing at random assumption. Imputation models were estimated separately for men and women and included:

1. All the baseline covariates used in the main analysis (alcohol consumption, age, quadratic age, socioeconomic deprivation, smoking status, systolic blood pressure, height and weight separately, whether provided dietary advice and diabetes status);

2. Baseline values of auxiliary variables not considered in the main analysis (ethnicity, number of consultations, diastolic blood pressure, pulse pressure, mean arterial pressure, high density lipoprotein cholesterol, total cholesterol, white cell count, haemoglobin, creatinine, glycated haemoglobin and gamma-glutamyl transferase)

3. Prior (between 1 and 4 years before study entry) averages of continuous covariates in the main analysis plus auxiliary variables;

4. Baseline medications (statins, antihypertensive medication, low-dose aspirin, loop diuretics, oral contraceptives and hormone replacement therapy);

5. Coexisting medical conditions (history of depression, anxiety, alcohol abuse, renal disease, dementia, liver disease or chronic obstructive pulmonary disease);

6. The Nelson-Aalen hazard and the event status for each endpoint analysed in the data.[90]

Non-normally distributed variables were log-transformed for imputation and exponentiated back to their original scale for analysis. Five multiply imputed datasets were generated, and Cox proportional hazard models were fitted to each dataset. Coefficients were combined using Rubin’s rules.[91] We checked whether the imputations were plausible by comparing plots of the distribution of observed and imputed values.

Distribution of initial presentations by drinking category

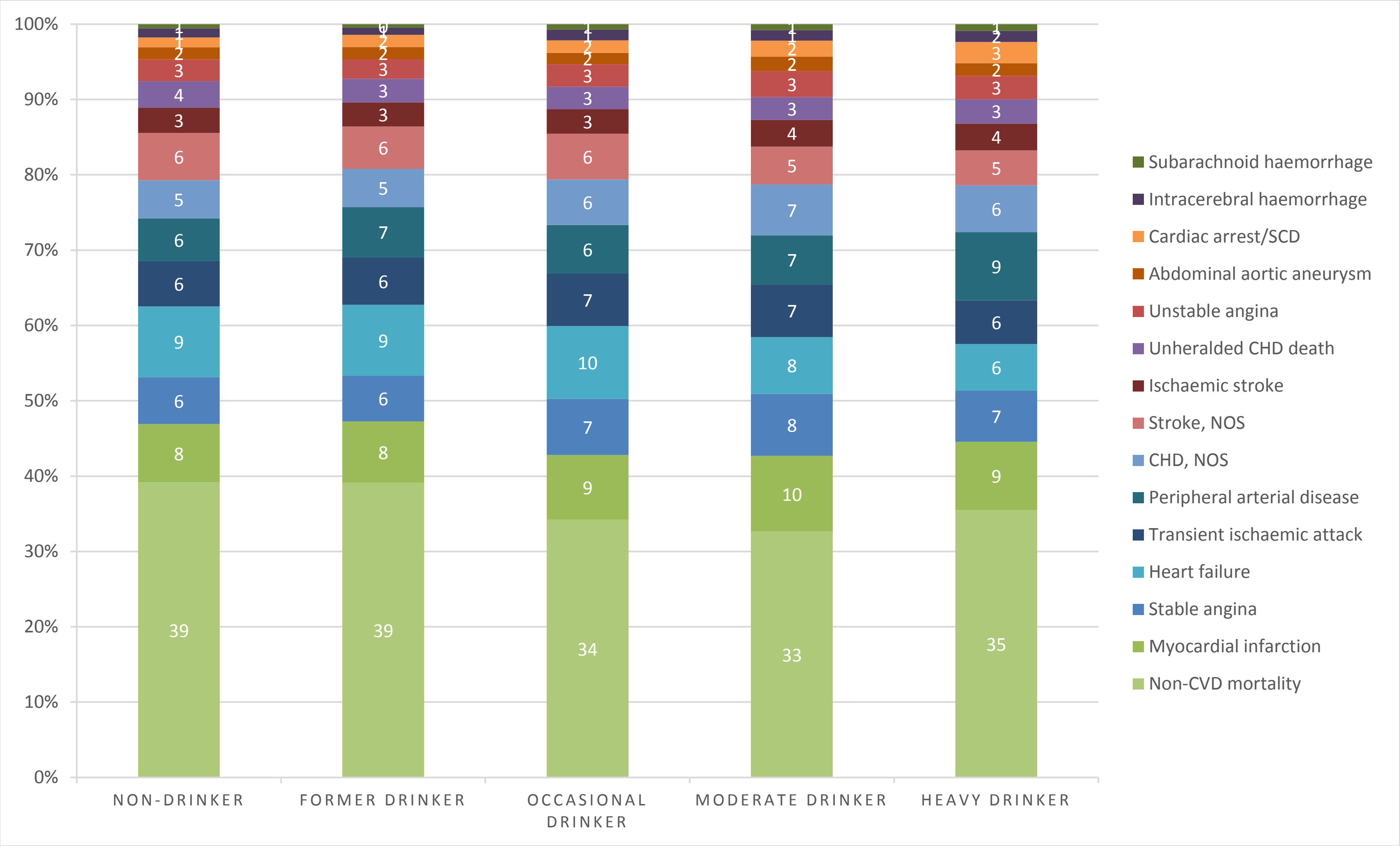

Figure D - Stacked bar chart of the distribution of 114,859 initial presentations by clinically recorded alcohol category amongst a cohort of 1.93 million adults

Hazard ratios for non-CVD mortality and, CHD and stroke, not otherwise specified

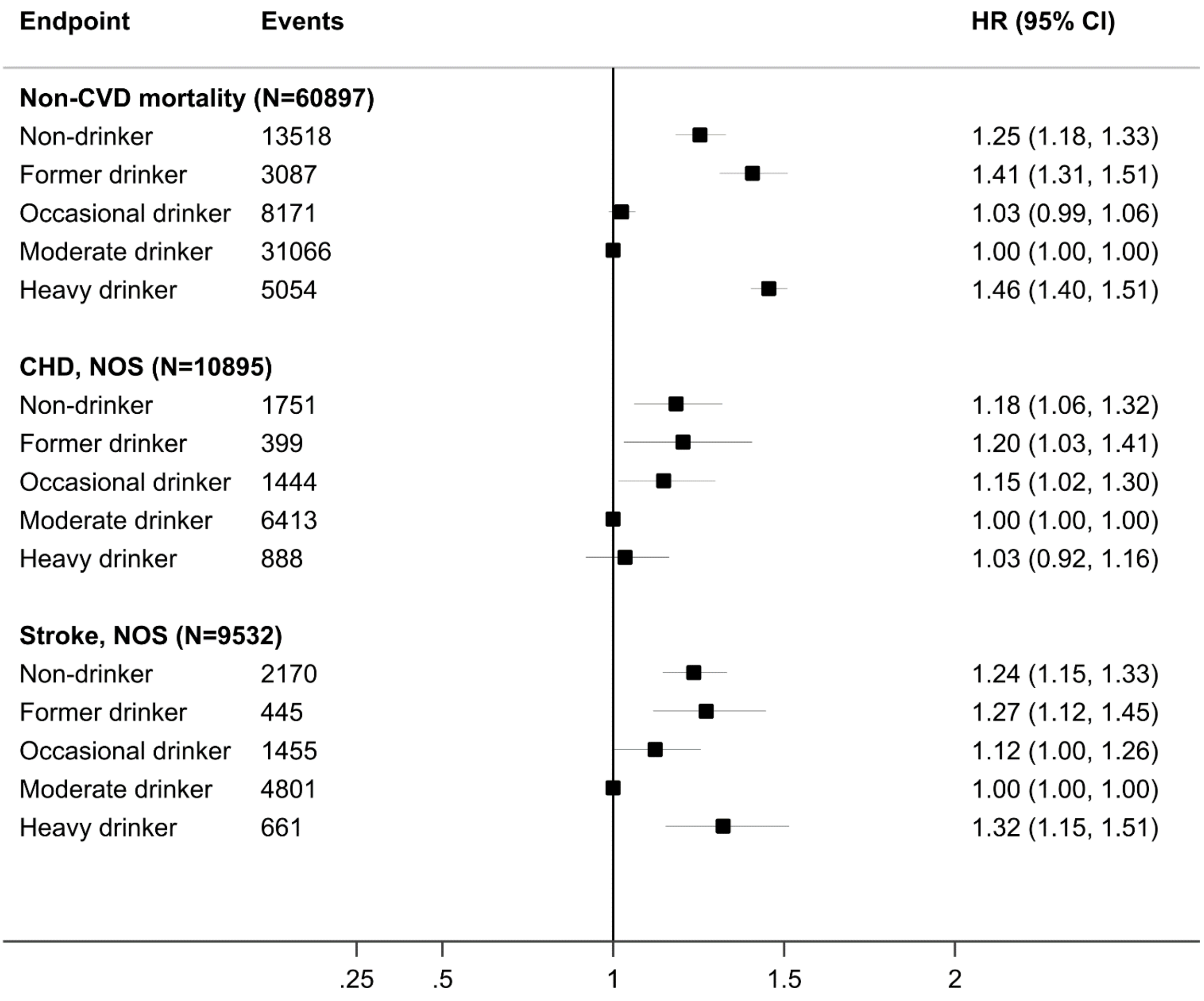

Figure E - Multivariable adjusted hazard ratios for non-CVD mortality and, CHD and stroke, not otherwise specified comparing non, former, occasional and hazardous drinkers with moderate drinkers in a cohort of 1.93 million adults

Hazard ratios for myocardial infarction subtypes

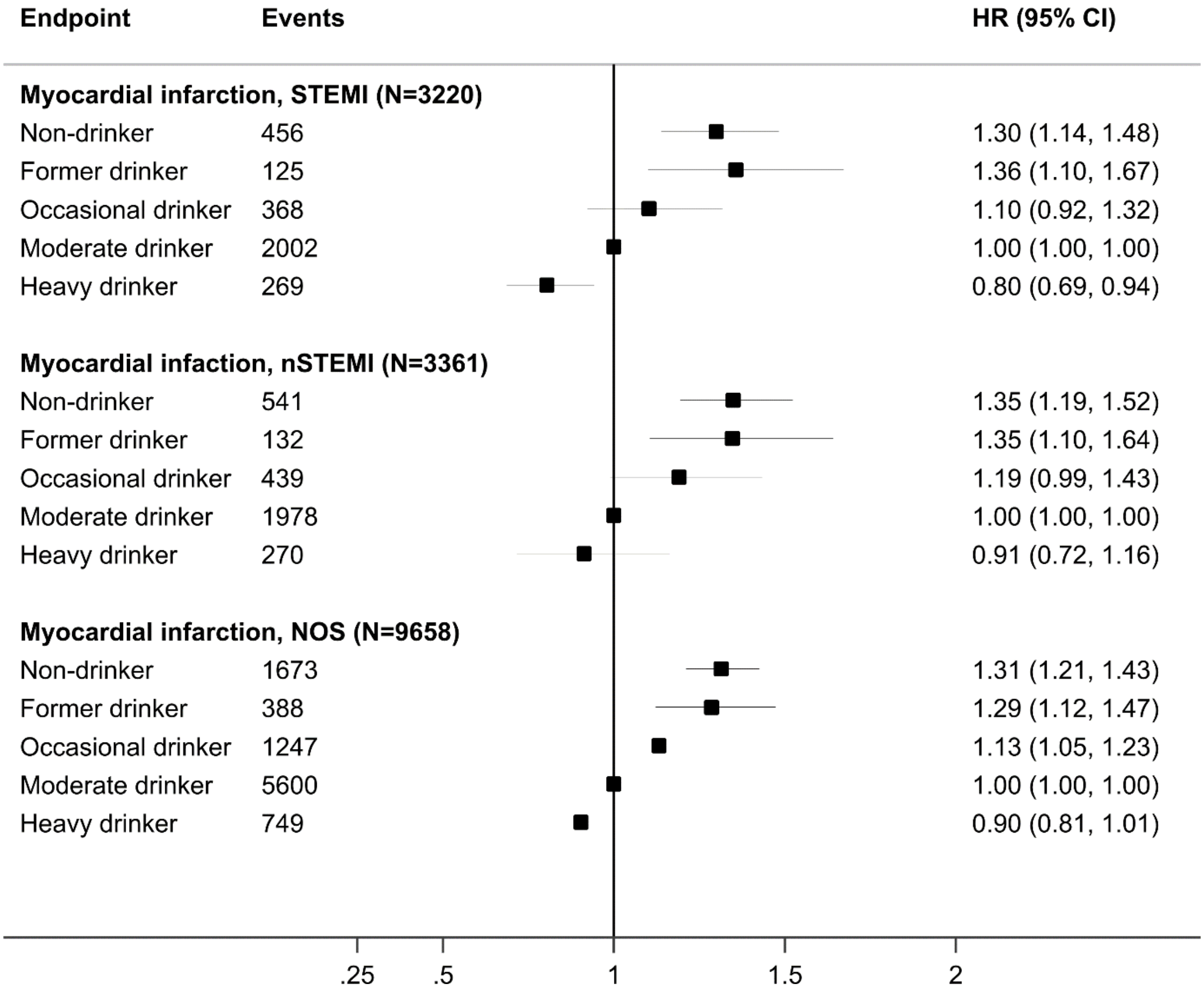

Figure F - Multivariable adjusted hazard ratios for myocardial infarction subtypes comparing non, former, occasional and hazardous drinkers with moderate drinkers in a cohort of 1.93 million adults

CI indicates confidence interval; STEMI, ST-elevated myocardial infarction; NSTEMI, non-ST-elevated myocardial infarction; NOS, not otherwise specified.

Tests of heterogeneity across CVD endpoints by drinking category

Table D - I^2 tests of heterogeneity in the association of drinking categories and 12 CVDs presented in the main analysis

Drinking category	I^2 (95% confidence interval)	p-value
Non-drinker	78% (62%, 87%)	< 0.001
Former drinker	42% (0%, 70%)	0.064
Occasional drinker	45% (9%, 72%)	0.05
Heavy drinker	86% (78%, 92%)	< 0.001

Supplementary analyses

We also calculated hazard ratios and 95% confidence intervals for the association of alcohol consumption and different CVDs adjusting for age and sex only.

In secondary analyses, we additionally adjusted for systolic blood pressure, diabetes mellitus status and body mass index which were not included as standard due to concerns of overadjustment bias given it is likely that they lie on the causal pathway between alcohol consumption and CVDs.[92]

We tested for effect modification by gender via including interactions between drinking category and sex for each endpoint.

In sensitivity analyses we re-analysed data (1) ignoring diagnoses obtained using primary care data, (2) limited to fatal endpoints only, and (3) using information collected after 2004 when financial incentives were introduced for recording patient data on alcohol consumption. We also compared findings obtained using imputed and complete case methods. We carried out a series of *post-hoc* analyses within subgroups defined by smoking status, BMI and diabetes.

Our referent category in all models was moderate drinkers.[93] All analyses, here and in the main paper, were conducted using Stata v14.

Adjusted for age and sex only

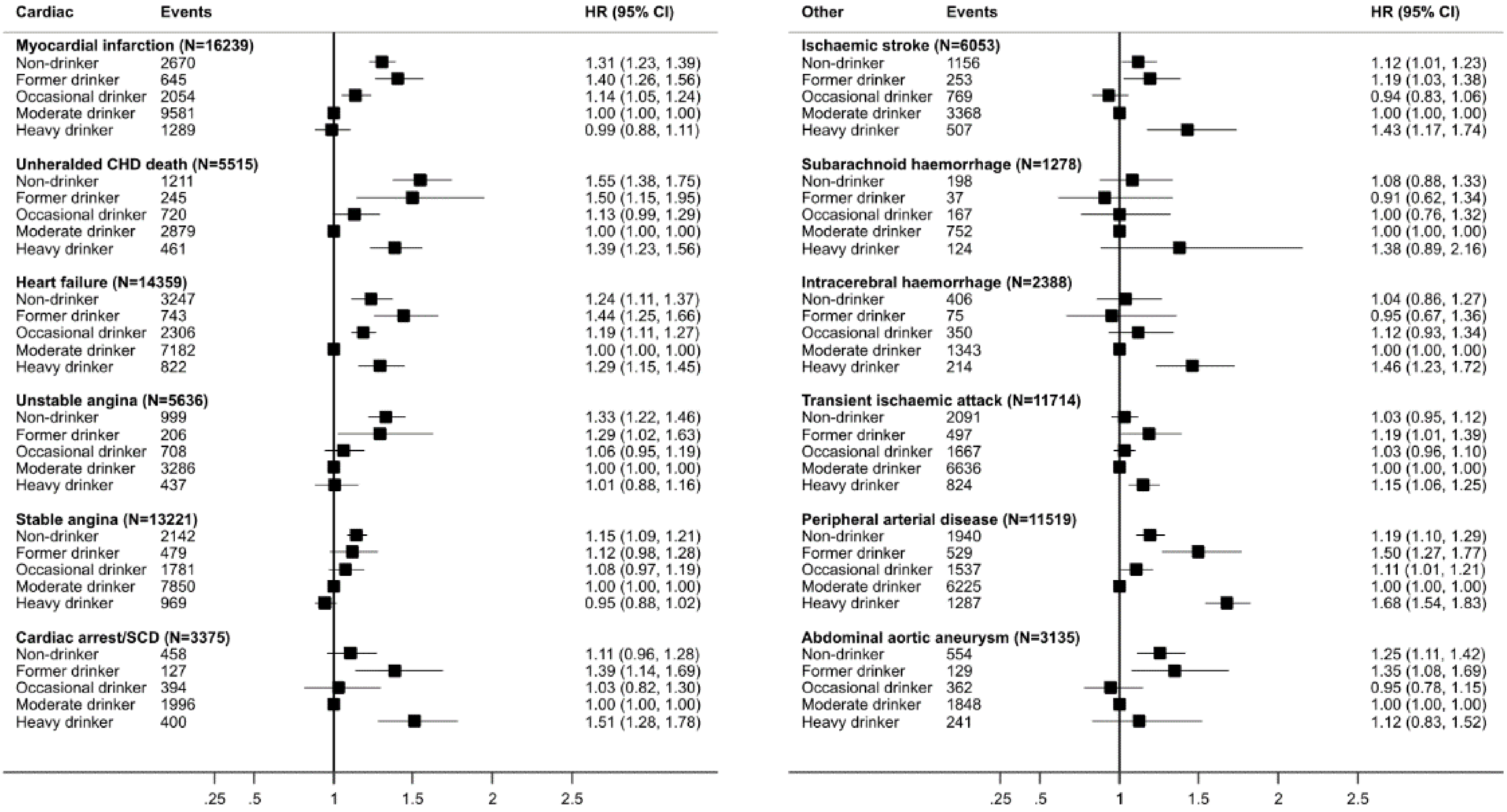

Figure G – Age and sex adjusted hazard ratios of 12 CVDs comparing non, former, occasional and heavy drinkers with moderate drinkers in a cohort of 1.93 million adults

Additional adjustment for systolic blood pressure, diabetes status, body mass index, HDL-cholesterol, use of statins or blood pressure lowering medication, and whether offered dietary advice

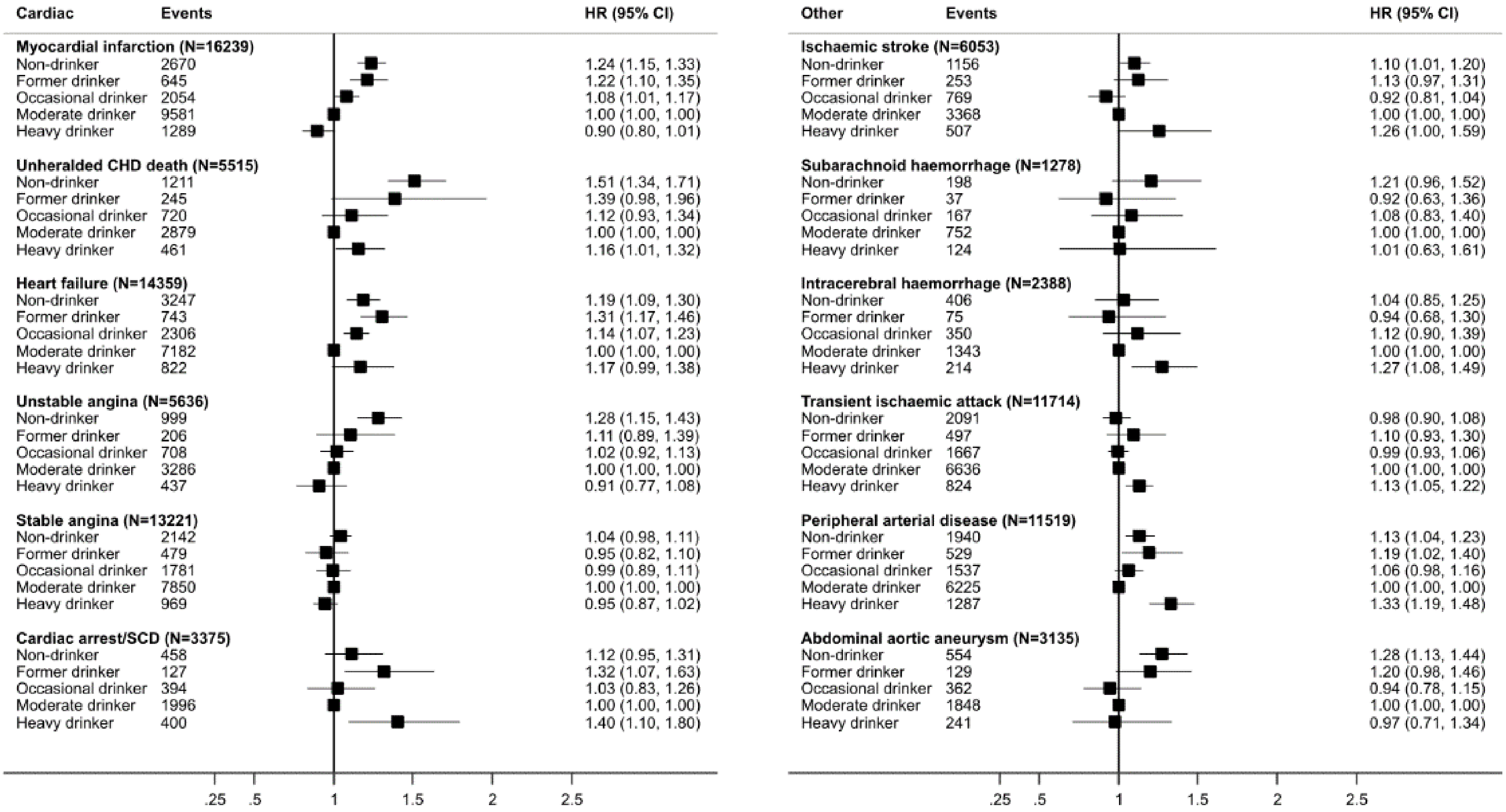

Figure H - Multivariable adjusted hazard ratios of 12 CVDs comparing non, former, occasional and heavy drinkers with moderate drinkers in a cohort of 1.93 million adults

Analyses by gender

P-values for interactions by sex

Table E - P-values for interaction between drinking categories and sex across all endpoints

Outcome	p-value
Stable angina	0.008
Unstable angina	0.1828
MI	0.0195
Unheralded CHD death	0.4048
Heart failure	0.0014
Cardiac arrest/SCD	0.729
Transient ischaemic attack	0.4054
Ischaemic stroke	0.0037
Subarachnoid haemorrhage	0.8122
Intracerebral haemorrhage	0.2258
Peripheral arterial disease	0.5539
Abdominal aortic aneurysm	0.8873
Non-CVD mortality	0.0005
CHD, NOS	0.0367
Stroke, NOS	0.0398

Only heart failure and non-CVD mortality meet the Bonferroni corrected significance p-value of 0.0033 (0.05/15). Analyses restricted to samples of the same sex are presented below in Figures I and J.

Main analyses limited to men

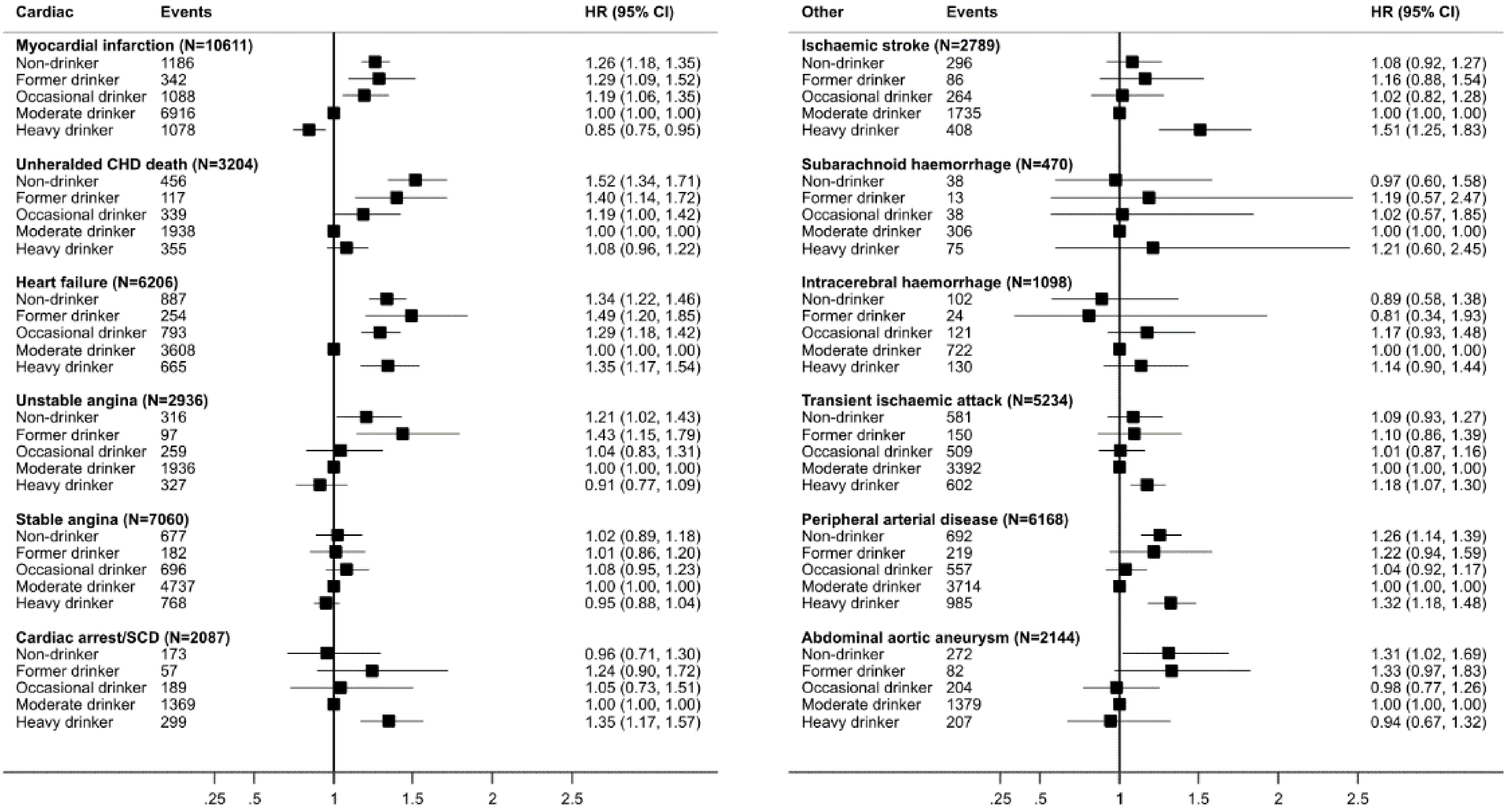

Figure I - Multivariable adjusted hazard ratios of 12 CVDs comparing non, former, occasional and heavy drinkers with moderate drinkers in a cohort of 958, 329 men

Main analyses limited to women

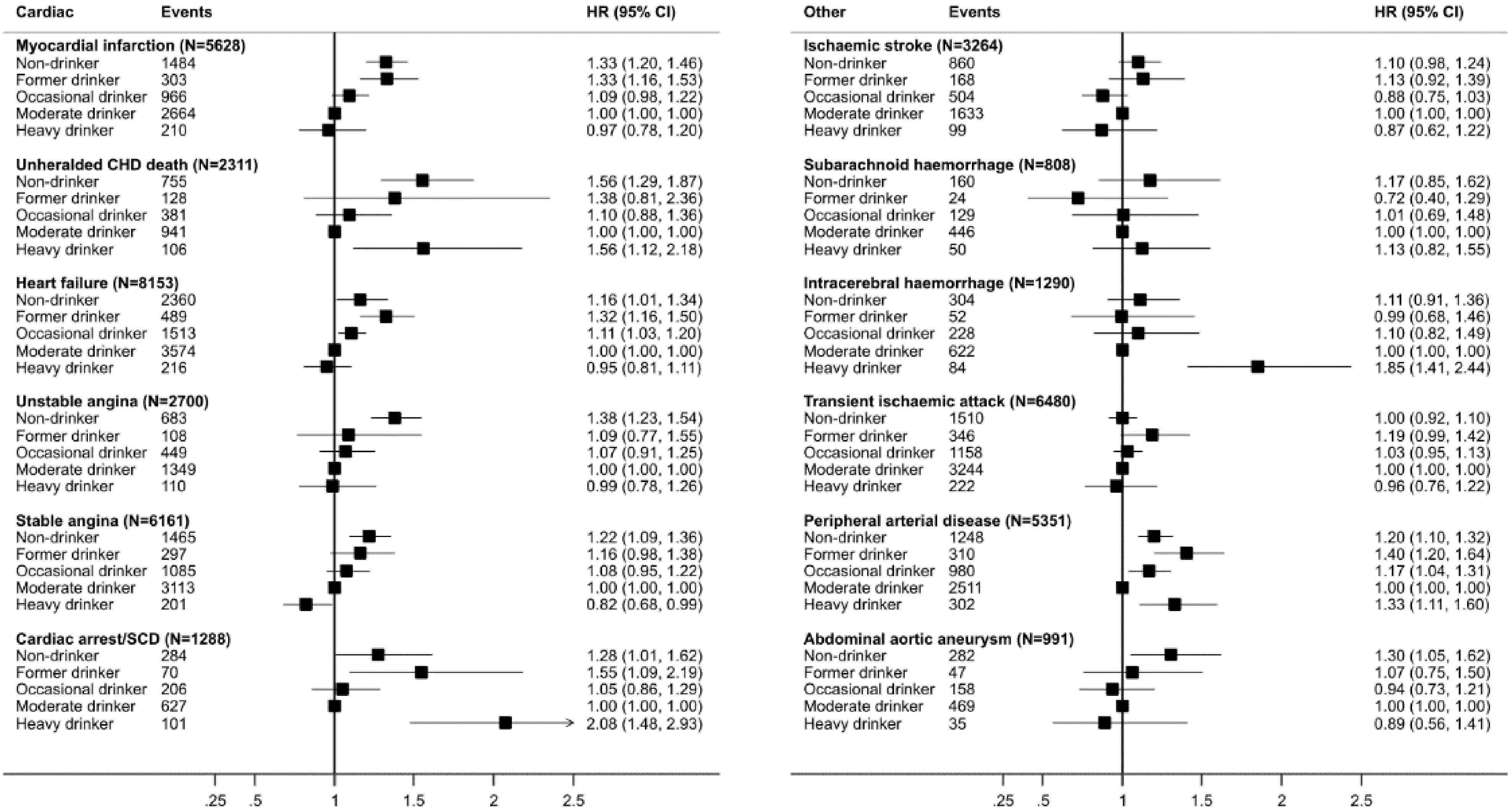

Figure J - Multivariable adjusted hazard ratios of 12 CVDs comparing non, former, occasional and heavy drinkers with moderate drinkers in a cohort of 979,031 women

Using only secondary care and mortality based endpoints

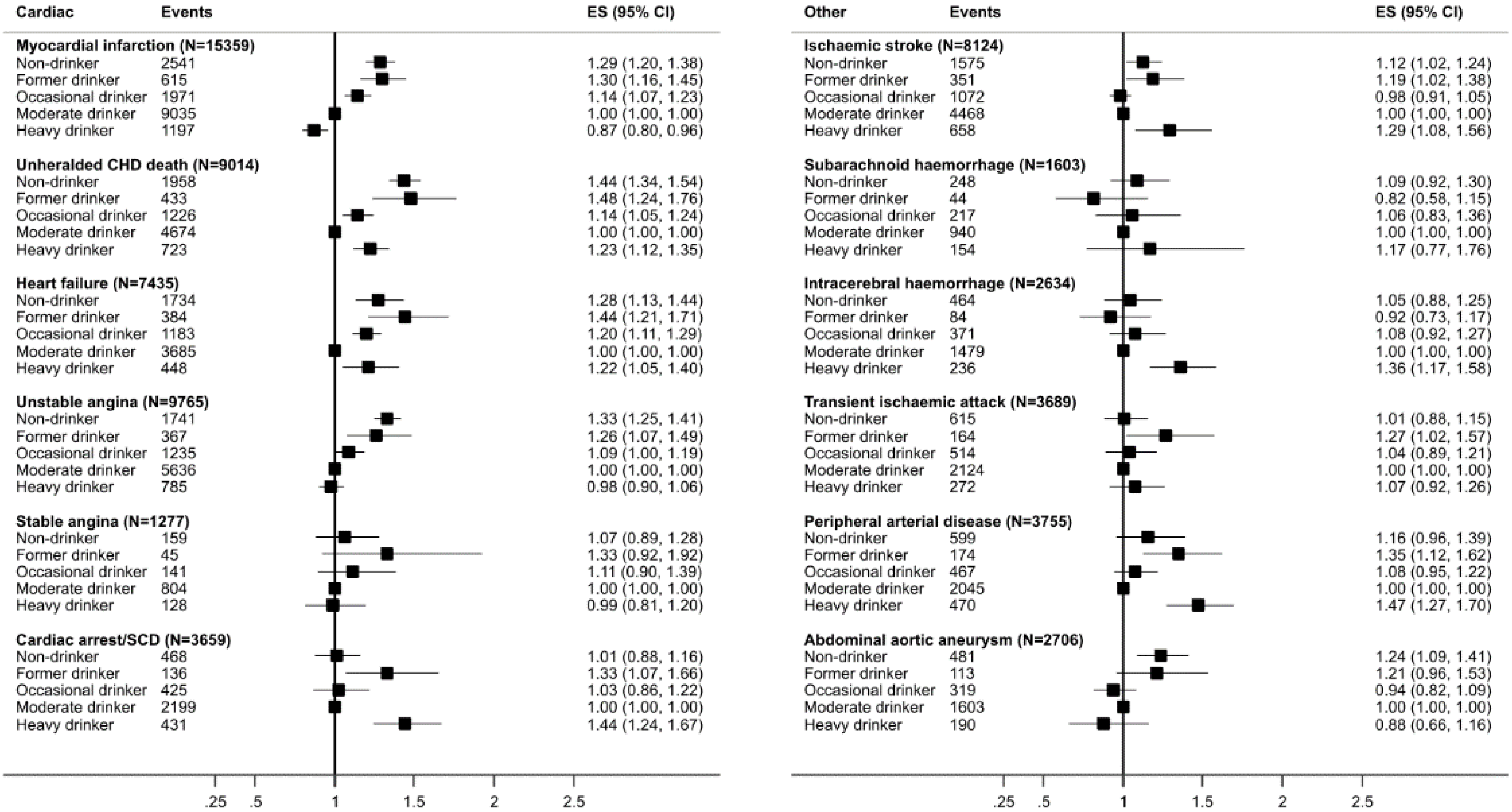

Figure K - Multivariable adjusted hazard ratios of 12 CVDs comparing non, former, occasional and heavy drinkers with moderate drinkers in a cohort of 1.93 million adults

Fatal endpoints only

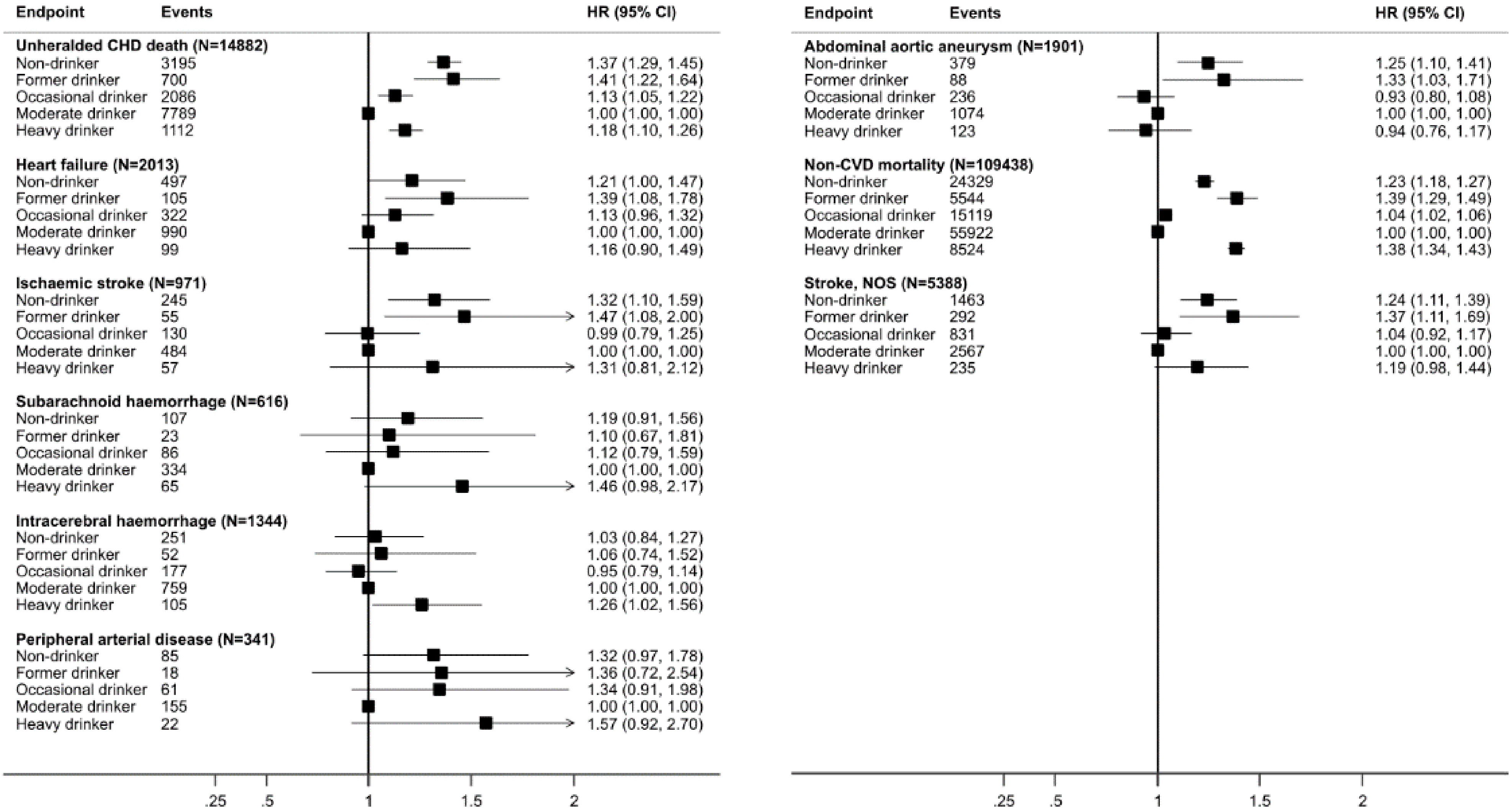

Figure L - Multivariable adjusted hazard ratios of 12 CVDs comparing non, former, occasional and heavy drinkers with moderate drinkers in a cohort of 1.93 million adults

Analyses using data collected post-2004

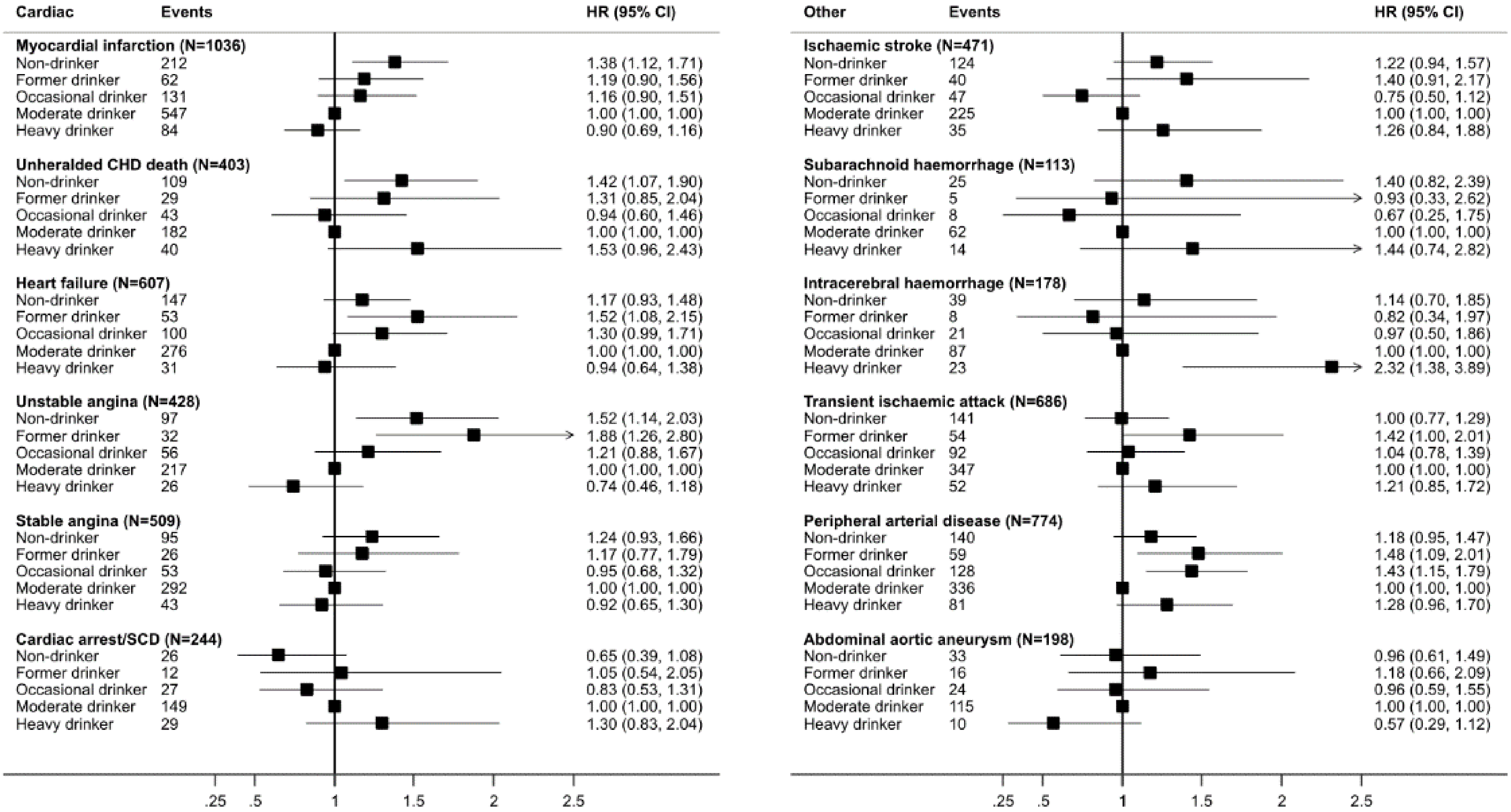

Figure M - Multivariable adjusted hazard ratios of 12 CVDs comparing non, former, occasional and heavy drinkers with moderate drinkers in a cohort of 597,027 adults

Complete case analysis

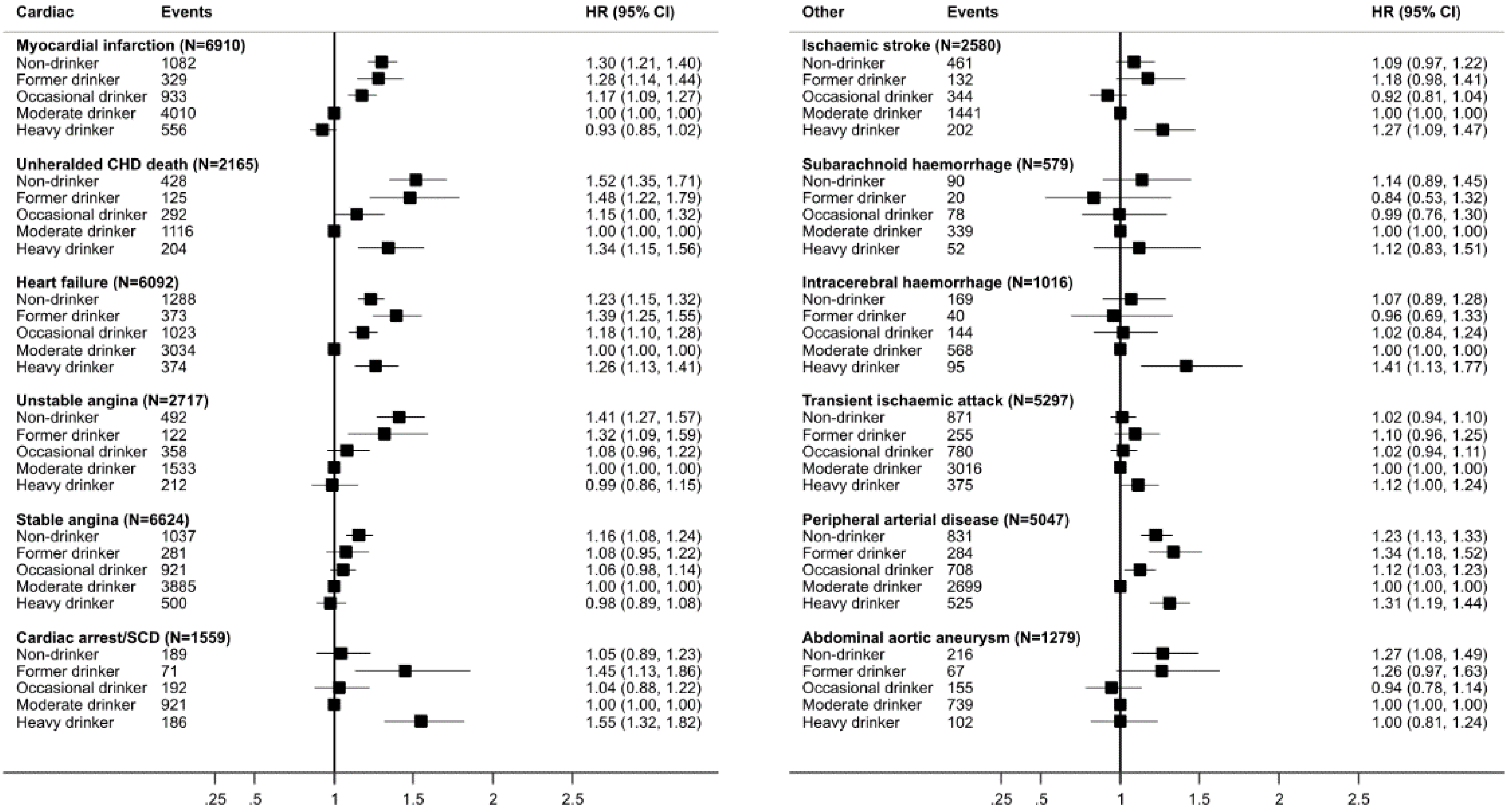

Figure N - Multivariable adjusted hazard ratios of 12 CVDs comparing non, former, occasional and heavy drinkers with moderate drinkers in a cohort of 1,009,578 adults

Analysis restricted to never smokers

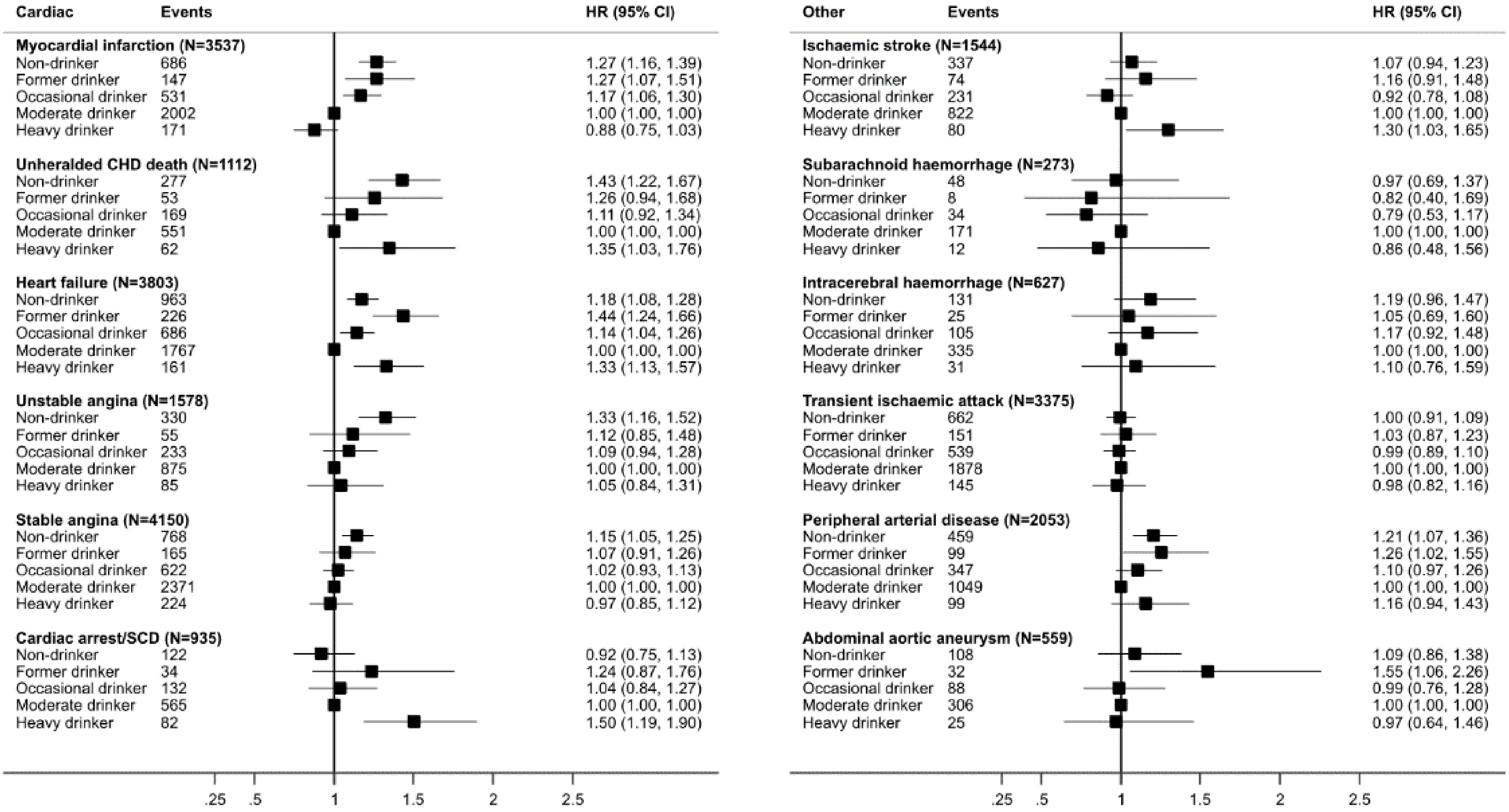

Figure O - Multivariable adjusted hazard ratios of 12 CVDs comparing non, former, occasional and heavy drinkers with moderate drinkers in a cohort of 599,148 adults with no history of smoking (observed data)

Analysis restricted to smokers

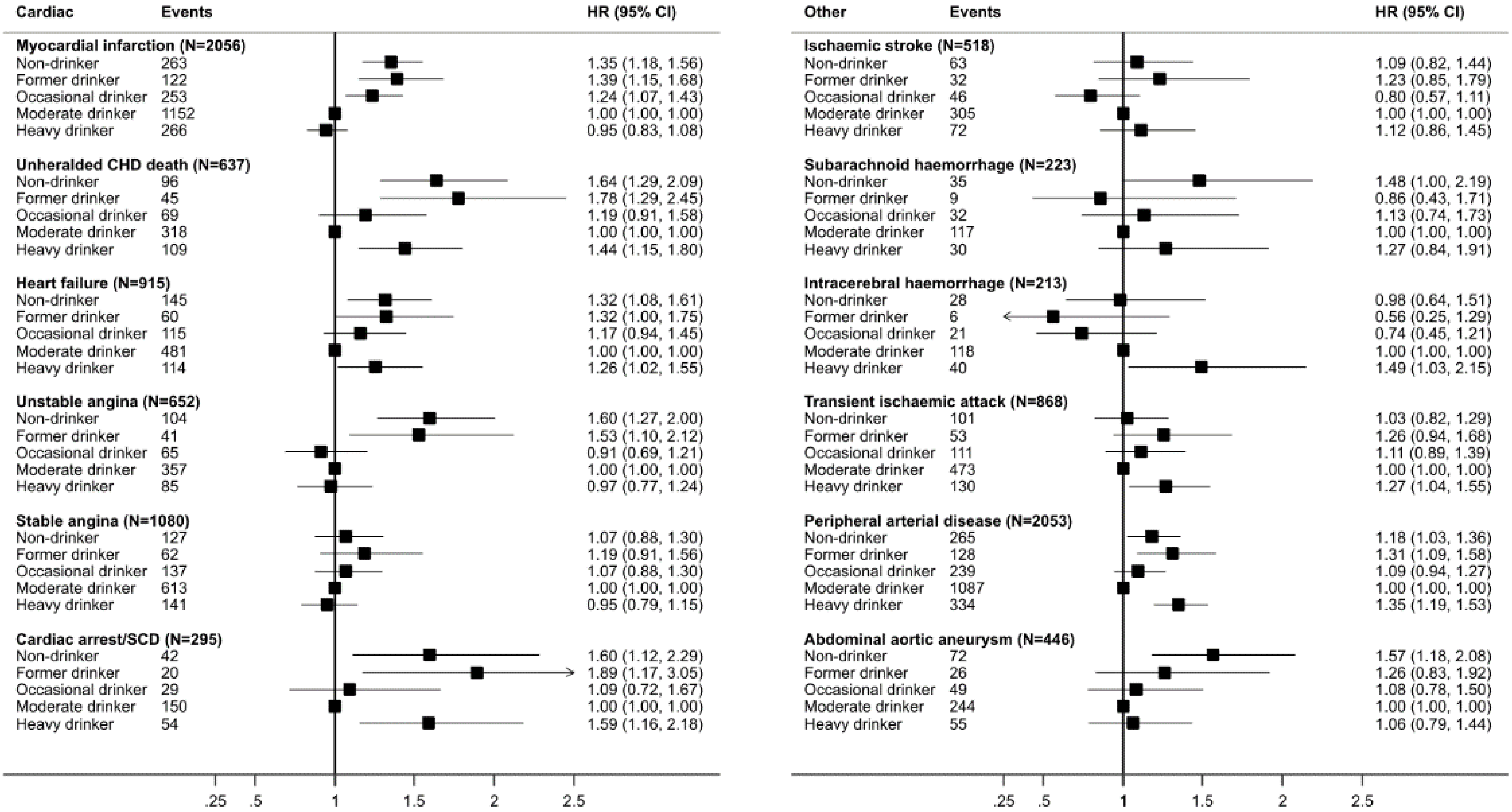

Figure P - Multivariable adjusted hazard ratios of 12 CVDs comparing non, former, occasional and heavy drinkers with moderate drinkers in a cohort of 234,942 current smoking adults (observed data)

Analysis restricted to those with BMI values in the normal range

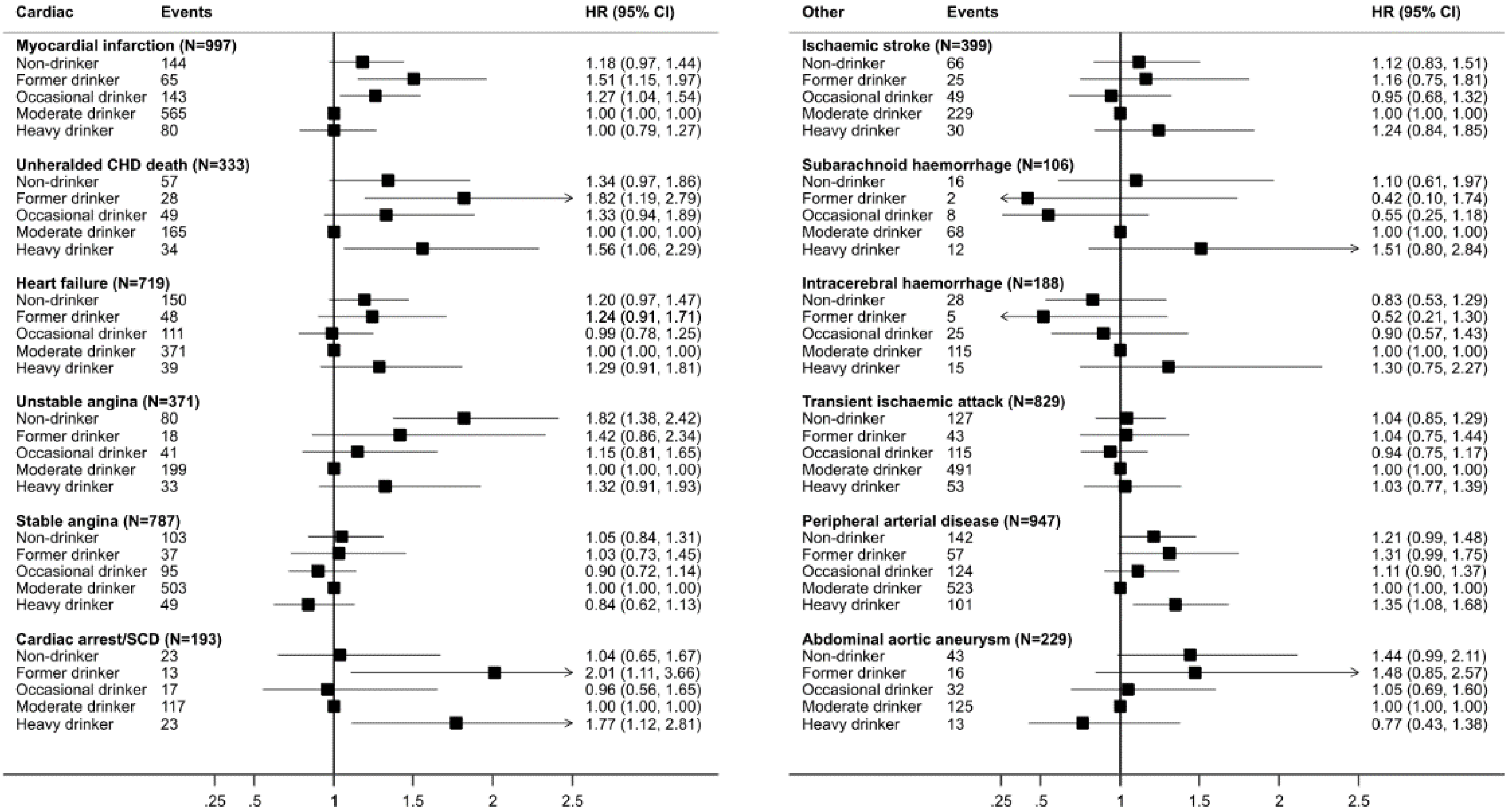

Figure Q - Multivariable adjusted hazard ratios of 12 CVDs comparing non, former, occasional and heavy drinkers with moderate drinkers in a cohort of 221,322 adults with a BMI in the normal range (observed data)

Analysis restricted to those with BMI values considered overweight

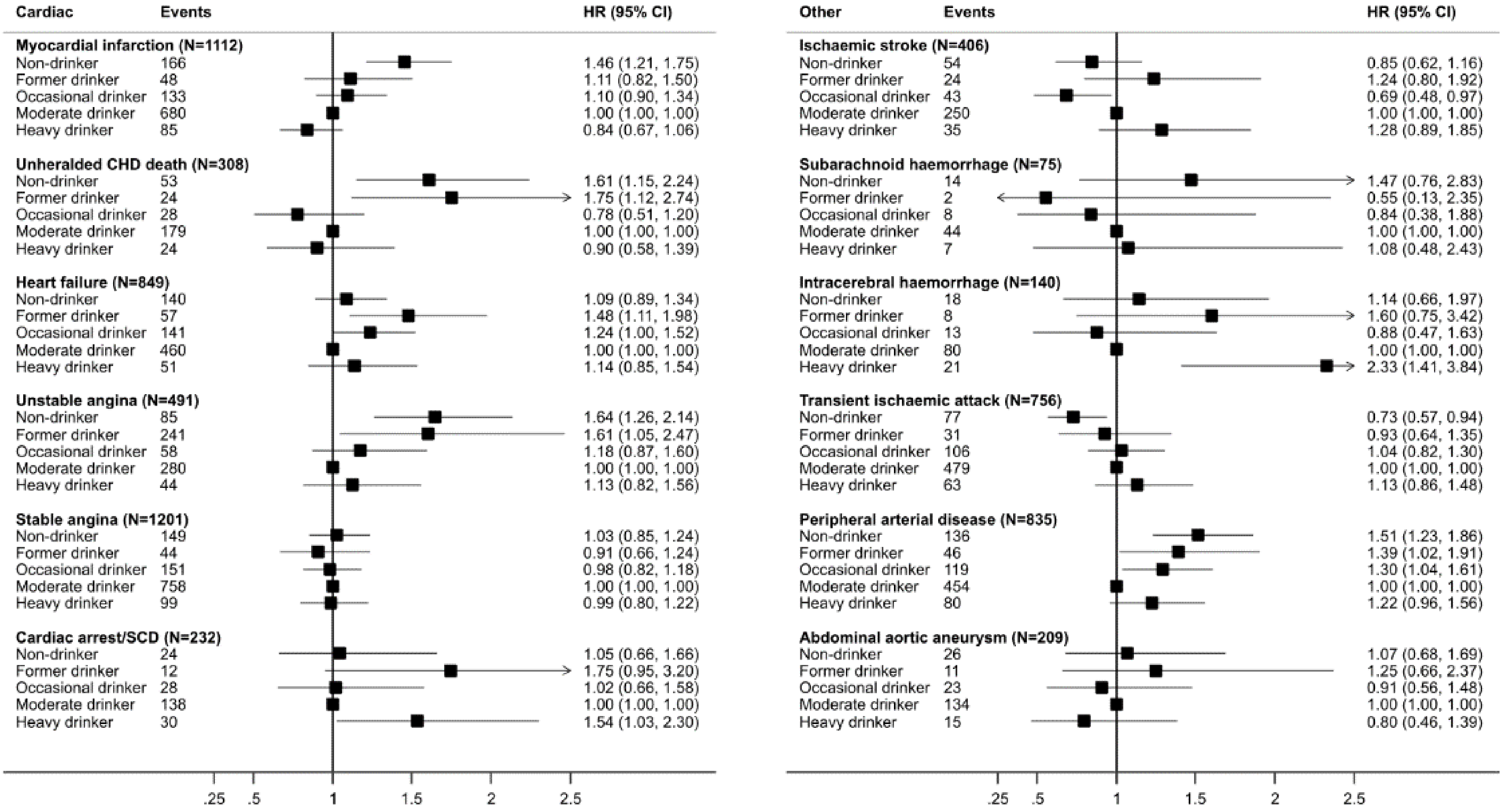

Figure R - Multivariable adjusted hazard ratios of 12 CVDs comparing non, former, occasional and heavy drinkers with moderate drinkers in a cohort of 178,413 adults with a BMI in the overweight range (observed data)

Analysis restricted to those with BMI values considered obese

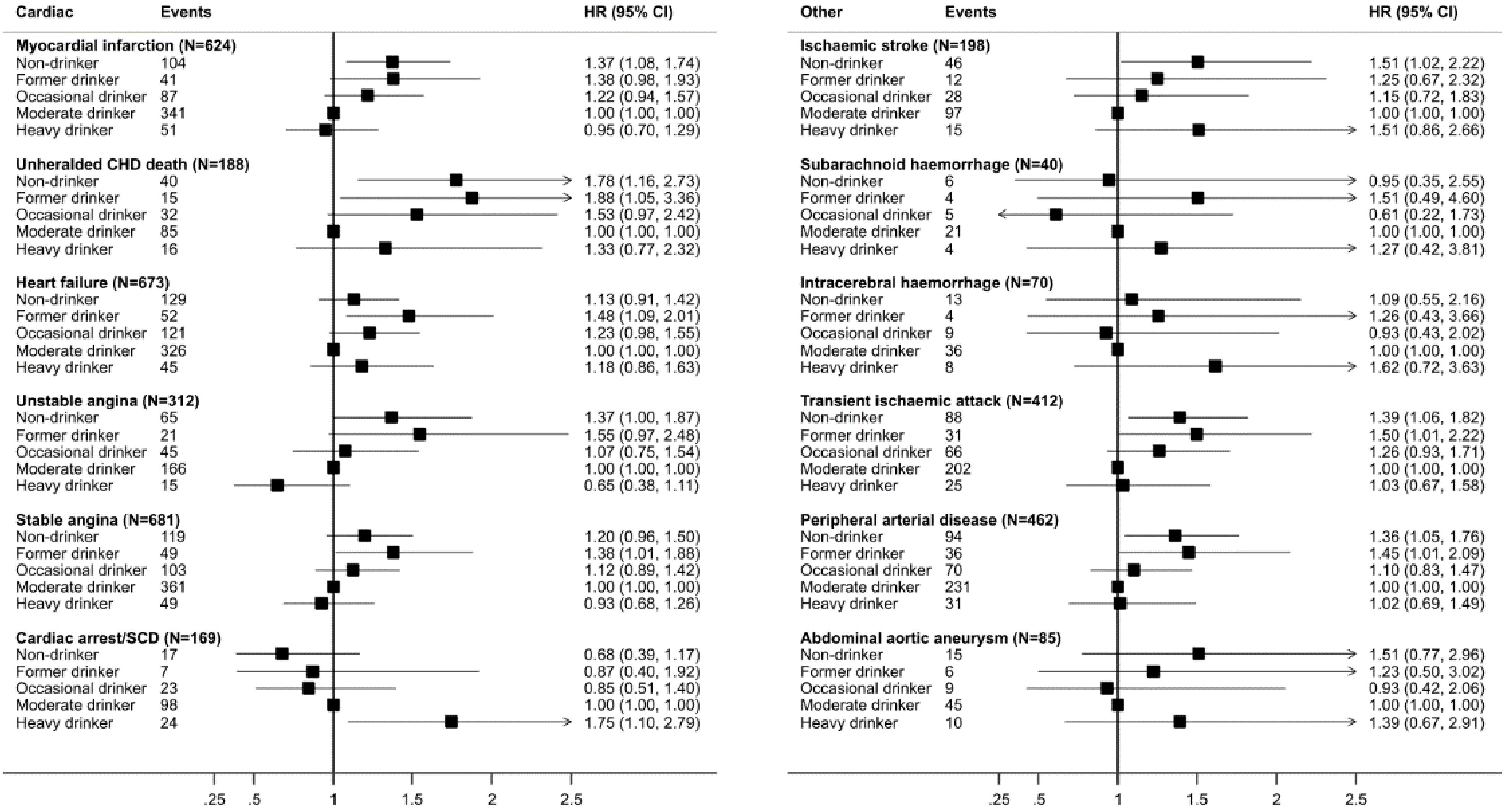

Figure S - Multivariable adjusted hazard ratios of 12 CVDs comparing non, former, occasional and heavy drinkers with moderate drinkers in a cohort of 97,692 adults with a BMI in the obese range (observed data)

Supplementary references

1. Roerecke M, Rehm J. The cardioprotective association of average alcohol consumption and ischaemic heart disease: a systematic review and meta-analysis. Addiction 2012;107(7):1246–60.

2. Roerecke M, Rehm J. Alcohol consumption, drinking patterns, and ischemic heart disease: a narrative review of meta-analyses and a systematic review and meta-analysis of the impact of heavy drinking occasions on risk for moderate drinkers. BMC Med 2014;12(1):182.

3. Ronksley PE, Brien SE, Turner BJ, Mukamal KJ, Ghali WA. Association of alcohol consumption with selected cardiovascular disease outcomes: a systematic review and meta-analysis. BMJ 2011;342:d671.

4. Fernández-Solà J. Cardiovascular risks and benefits of moderate and heavy alcohol consumption. Nat Rev Cardiol 2015;12(10):576–87.

5. Movva R, Figueredo VM. Alcohol and the heart: To abstain or not to abstain? Int J Cardiol 2013;164(3):267–76.

6. O'Keefe JH, Bybee KA, Lavie CJ. Alcohol and Cardiovascular Health: The Razor-Sharp Double-Edged Sword. J Am Coll Cardiol 2007;50(11):1009–14.

7. Mukamal K, Rimm E. Alcohol consumption: Risks and benefits. Curr Atheroscler Rep 2008;10(6):536–43.

8. Brien SE, Ronksley PE, Turner BJ, Mukamal KJ, Ghali WA. Effect of alcohol consumption on biological markers associated with risk of coronary heart disease: systematic review and meta-analysis of interventional studies. BMJ 2011;342:d636.

9. Mathews MJ, Liebenberg L, Mathews EH. The mechanism by which moderate alcohol consumption influences coronary heart disease. Nutr J 2015;14:33.

10. Chikritzhs T, Stockwell T, Naimi T, Andreasson S, Dangardt F, Liang W. Has the leaning tower of presumed health benefits from "moderate" alcohol use finally collapsed? Addiction 2015;110(5):726–7.

11. Fekjær HO. Alcohol—a universal preventive agent? A critical analysis. Addiction 2013;108(12):2051–7.

12. Stockwell T, Greer A, Fillmore K, Chikritzhs T, Zeisser C. How good is the science? BMJ 2012;344:e2276.

13. Roerecke M, Rehm J. Ischemic Heart Disease Mortality and Morbidity Rates in Former Drinkers: A Meta-Analysis. Am J Epidemiol 2011;173(3):245–58.

14. Ng Fat L, Cable N, Shelton N. Worsening of Health and a Cessation or Reduction in Alcohol Consumption to Special Occasion Drinking Across Three Decades of the Life Course. Alcohol Clin Exp Res 2015;39(1):166–74.

15. Holmes MV, Asselbergs FW, Palmer TM, et al. Mendelian randomization of blood lipids for coronary heart disease. Eur Heart J 2014;36:539–50.

16. Barter PJ, Caulfield M, Eriksson M, et al. Effects of Torcetrapib in Patients at High Risk for Coronary Events. N Engl J Med 2007;357(21):2109–22.

17. Yaghootkar H, Lamina C, Scott RA, et al. Mendelian Randomisation Studies Do Not Support a Causal Role for Reduced Circulating Adiponectin Levels in Insulin Resistance and Type 2 Diabetes. Diabetes 2013;62(10):3589–98.

18. Keavney B, Danesh J, Parish S, et al. Fibrinogen and coronary heart disease: test of causality by “Mendelian randomization.” Int J Epidemiol 2006;35(4):935–43.

19. Sook Lee E, Park S, Kim E, et al. Association between adiponectin levels and coronary heart disease and mortality: a systematic review and meta-analysis. Int J Epidemiol 2013;42(4):1029–39.

20. Holmes MV, Dale CE, Zuccolo L, et al. Association between alcohol and cardiovascular disease: Mendelian randomisation analysis based on individual participant data. BMJ 2014;349:g4164.

21. Silverwood RJ, Holmes MV, Dale CE, et al. Testing for non-linear causal effects using a binary genotype in a Mendelian randomization study: application to alcohol and cardiovascular traits. Int J Epidemiol 2014;43(6):1781–90.

22. Mukamal K. Alcohol Intake and Noncoronary Cardiovascular Diseases. Ann Epidemiol 2007;17(5, Supplement):S8–12.

23. Breslow RA, Mukamal KJ. Measuring the Burden—Current and Future Research Trends: Results From the NIAAA Expert Panel on Alcohol and Chronic Disease Epidemiology. Alcohol Res Curr Rev 2014;35(2):250.

24. Stockwell T, Zhao J, Panwar S, Roemer A, Naimi T, Chikritzhs T. Do “moderate” drinkers have reduced mortality risk? A systematic review and meta-analysis of alcohol consumption and all-cause mortality. J Stud Alcohol Drugs 2016;77(2):185–98.

25. Camargo J Carlos A, Stampfer MJ, Glynn RJ, et al. Moderate Alcohol Consumption and Risk for Angina Pectoris or Myocardial Infarction in U.S. Male Physicians. Ann Intern Med 1997;126(5):372–5.

26. Merry AH, Boer JM, Schouten LJ, et al. Smoking, alcohol consumption, physical activity, and family history and the risks of acute myocardial infarction and unstable angina pectoris: a prospective cohort study. BMC Cardiovasc Disord 2011;11(1):1–14.

27. Hange D, Sigurdsson JA, Björkelund C, Sundh V, Bengtsson C. A 32-year longitudinal study of alcohol consumption in Swedish women: Reduced risk of myocardial infarction but increased risk of cancer. Scand J Prim Health Care 2015;33(3):153–62.

28. Gémes K, Janszky I, Laugsand LE, et al. Alcohol consumption is associated with a lower incidence of acute myocardial infarction: results from a large prospective population-based study in Norway. J Intern Med 2016;279(4):365–75.

29. Smyth A, Teo KK, Rangarajan S, et al. Alcohol consumption and cardiovascular disease, cancer, injury, admission to hospital, and mortality: a prospective cohort study. The Lancet 14;386(10007):1945–54.

30. Romelsjö A, Allebeck P, Andréasson S, Leifman A. Alcohol, Mortality and Cardiovascular Events in a 35 Year Follow-up of a Nationwide Representative Cohort of 50,000 Swedish Conscripts up to Age 55. Alcohol Alcohol 2012;47(3):322–7.

31. Maclure M. Demonstration of Deductive Meta-Analysis: Ethanol Intake and Risk of Myocardial Infarction. Epidemiol Rev 1993;15(2):328–51.

32. Cleophas TJ. Wine, beer and spirits and the risk of myocardial infarction: a systematic review. Biomed Pharmacother 1999;53(9):417–23.

33. Klatsky AL, Chartier D, Udaltsova N, et al. Alcohol Drinking and Risk of Hospitalization for Heart Failure With and Without Associated Coronary Artery Disease. Am J Cardiol 2005;96(3):346–51.

34. Djoussé L, Gaziano JM. Alcohol Consumption and Risk of Heart Failure in the Physicians' Health Study I. Circulation 2007;115(1):34–9.

35. Wang Y, Tuomilehto J, Jousilahti P, et al. Lifestyle Factors in Relation to Heart Failure Among Finnish Men and Women. Circ Heart Fail 2011;4(5):607–12.

36. Larsson SC, Orsini N, Wolk A. Alcohol consumption and risk of heart failure: a dose–response meta-analysis of prospective studies. Eur J Heart Fail 2015;17(4):367–73.

37. Padilla H, Gaziano JM, Djoussé L. Alcohol Consumption and Risk of Heart Failure: A Meta-Analysis. Phys Sportsmed 2010;38(3):84–9.

38. Chiuve SE, Rimm EB, Mukamal KJ, et al. Light-to-moderate alcohol consumption and risk of sudden cardiac death in women. Heart Rhythm 2010;7(10):1374–80.

39. Albert CM, Manson JE, Cook NR, Ajani UA, Gaziano JM, Hennekens CH. Moderate Alcohol Consumption and the Risk of Sudden Cardiac Death Among US Male Physicians. Circulation 1999;100(9):944–50.

40. Weikert C, Berger K, Heidemann C, et al. Joint effects of risk factors for stroke and transient ischemic attack in a German population. J Neurol 2007;254(3):315–21.

41. Stampfer MJ, Colditz GA, Willett WC, Speizer FE, Hennekens CH. A Prospective Study of Moderate Alcohol Consumption and the Risk of Coronary Disease and Stroke in Women. N Engl J Med 1988;319(5):267–73.

42. Iso H, Baba S, Mannami T, et al. Alcohol Consumption and Risk of Stroke Among Middle-Aged Men: The JPHC Study Cohort I. Stroke 2004;35(5):1124–9.

43. Klatsky AL, Armstrong MA, Friedman GD. Alcohol use and subsequent cerebrovascular disease hospitalizations. Stroke 1989;20(6):741–6.

44. Mukamal KJ, Ascherio A, Mittleman MA, et al. Alcohol and Risk for Ischemic Stroke in Men: The Role of Drinking Patterns and Usual Beverage. Ann Intern Med 2005;142(1):11–9.

45. Nielsen NR, Truelsen T, Barefoot JC, et al. Is the Effect of Alcohol on Risk of Stroke Confined to Highly Stressed Persons? Neuroepidemiology 2005;25(3):105–13.

46. Lu M, Ye W, Adami H-O, Weiderpass E. Stroke Incidence in Women under 60 Years of Age Related to Alcohol Intake and Smoking Habit. Cerebrovasc Dis 2008;25(6):517–25.

47. Ikehara S, Iso H, Toyoshima H, et al. Alcohol Consumption and Mortality From Stroke and Coronary Heart Disease Among Japanese Men and Women: The Japan Collaborative Cohort Study. Stroke 2008;39(11):2936–42.

48. Bos S, Grobbee DE, Boer JMA, Verschuren WM, Beulens JWJ. Alcohol consumption and risk of cardiovascular disease among hypertensive women. Eur J Cardiovasc Prev Rehabil 2010;17(1):119–26.

49. Kadlecová P, Andel R, Mikulík R, Handing EP, Pedersen NL. Alcohol Consumption at Midlife and Risk of Stroke During 43 Years of Follow-Up: Cohort and Twin Analyses. Stroke 2015;46(3):627–33.

50. Jones SB, Loehr L, Avery CL, et al. Midlife Alcohol Consumption and the Risk of Stroke in the Atherosclerosis Risk in Communities Study. Stroke 2015;46(11):3124–30.

51. Zhang C, Qin Y-Y, Chen Q, et al. Alcohol intake and risk of stroke: A dose–response meta-analysis of prospective studies. Int J Cardiol 2014;174(3):669–77.

52. Patra J, Taylor B, Irving H, et al. Alcohol consumption and the risk of morbidity and mortality for different stroke types - a systematic review and meta-analysis. BMC Public Health 2010;10(1):258.

53. Reynolds K, Lewis B, Nolen JDL, Kinney GL, Sathya B, He J. Alcohol consumption and risk of stroke: a meta-analysis. JAMA 2003;289(5):579–88.

54. Lindekleiv H, Sandvei MS, Njølstad I, et al. Sex differences in risk factors for aneurysmal subarachnoid hemorrhage: A cohort study. Neurology 2011;76(7):637–43.

55. Krishna V, Kim DH. Ethnic differences in risk factors for subarachnoid hemorrhage. J Neurosurg 2007;107(3):522–9.

56. Yao X, Zhang K, Bian J, Chen G. Alcohol consumption and risk of subarachnoid hemorrhage: A meta-analysis of 14 observational studies. Biomed Rep 2016;5:428–36.

57. Larsson SC, Wallin A, Wolk A, Markus HS. Differing association of alcohol consumption with different stroke types: a systematic review and meta-analysis. BMC Med 2016;14(1):178.

58. Ariesen MJ, Claus SP, Rinkel GJE, Algra A. Risk Factors for Intracerebral Hemorrhage in the General Population: A Systematic Review. Stroke 2003;34(8):2060–5.

59. Camargo CA, Stampfer MJ, Glynn RJ, et al. Prospective Study of Moderate Alcohol Consumption and Risk of Peripheral Arterial Disease in US Male Physicians. Circulation 1997;95(3):577–80.

60. Stackelberg O, Björck M, Larsson SC, Orsini N, Wolk A. Alcohol Consumption, Specific Alcoholic Beverages, and Abdominal Aortic Aneurysm. Circulation 2014;130(8):646–52.

61. Wong DR, Willett WC, Rimm EB. Smoking, Hypertension, Alcohol Consumption, and Risk of Abdominal Aortic Aneurysm in Men. Am J Epidemiol 2007;165(7):838–45.

62. Törnwall ME, Virtamo J, Haukka JK, Albanes D, Huttunen JK. Life-Style Factors and Risk for Abdominal Aortic Aneurysm in a Cohort of Finnish Male Smokers. Epidemiology [Internet] 2001;12(1). Available from: http://journals.lww.com/epidem/Fulltext/2001/01000/Life_Style_Factors_and_Risk_for_Abdominal_Aortic.16.aspx

63. Denaxas SC, George J, Herrett E, et al. Data Resource Profile: Cardiovascular disease research using linked bespoke studies and electronic health records (CALIBER). Int J Epidemiol 2012;41(6):1625–38.

64. Walley T, Mantgani A. The UK General Practice Research Database. The Lancet 1997;350(9084):1097–9.

65. Herrett E, Smeeth L, Walker L, Weston C, on behalf of the MINAP Academic Group. The Myocardial Ischaemia National Audit Project (MINAP). Heart 2010;96(16):1264–7.

66. Chisholm J. The Read clinical classification. BMJ 1990;300(6732):1092–1092.

67. World Health Organisation. International Classification of Diseases and Related Health Problems, Tenth Revision (ICD-10). Vols 1-3. Fourth Edition. Geneva: World Health Organisation; 1992.

68. National Health Service. OPCS-4 Classification - NHS Connecting for Health. National Health Service; 2013.

69. Department of Health. Sensible drinking: Report of an inter-departmental working group [Internet]. London: Department of Health; 1995 [cited 2009 Dec 19]. Available from: http://www.dh.gov.uk/prod_consum_dh/groups/dh_digitalassets/@dh/@en/documents/digitalasset/dh_4084702.pdf

70. Shaper AG, Wannamethee G, Walker M. Alcohol and mortality in British men: explaining the U-shaped curve. The Lancet 1988;332(8623):1267–73.

71. Ng Fat L, Shelton N. Associations between self-reported illness and non-drinking in young adults. Addiction 2012;107(9):1612–20.

72. Liu Y, Wang K, Maisonet M, Wang L, Zheng S. Associations of lifestyle factors (smoking, alcohol consumption, diet and physical activity) with type 2 diabetes among American adults from National Health and Nutrition Examination Survey (NHANES) 2005–2014. J Diabetes 2016;n/a-n/a.

73. Inada S, Koga M. Alcohol consumption reduces HbA1c and glycated albumin concentrations but not 1,5-anhydroglucitol. Ann Clin Biochem Int J Biochem Lab Med [Internet] 2016;Available from: http://acb.sagepub.com/content/early/2016/11/02/0004563216675646.abstract

74. Koning SH, Gansevoort RT, Mukamal KJ, et al. Alcohol consumption is inversely associated with the risk of developing chronic kidney disease. Kidney Int 2015;87(5):1009–16.

75. Bobak M, Malyutina S, Horvat P, et al. Alcohol, drinking pattern and all-cause, cardiovascular and alcohol-related mortality in Eastern Europe. Eur J Epidemiol 2016;31(1):21–30.

76. Herrett E, Shah AD, Boggon R, et al. Completeness and diagnostic validity of recording acute myocardial infarction events in primary care, hospital care, disease registry, and national mortality records: cohort study. BMJ 2013;346:f2350.

77. Payne RA, Abel GA, Simpson CR. A retrospective cohort study assessing patient characteristics and the incidence of cardiovascular disease using linked routine primary and secondary care data. BMJ Open [Internet] 2012;2(2). Available from: http://bmjopen.bmj.com/content/2/2/e000723.abstract

78. Burns EM, Rigby E, Mamidanna R, et al. Systematic review of discharge coding accuracy. J Public Health 2012;34(1):138–48.

79. Herrett E, Thomas SL, Schoonen WM, Smeeth L, Hall AJ. Validation and validity of diagnoses in the General Practice Research Database: a systematic review. Br J Clin Pharmacol 2010;69(1):4–14.

80. Kappert K, Böhm M, Schmieder R, et al. Impact of Sex on Cardiovascular Outcome in Patients at High Cardiovascular Risk: Analysis of the Telmisartan Randomized Assessment Study in ACE-

Intolerant Subjects With Cardiovascular Disease (TRANSCEND) and the Ongoing Telmisartan Alone and in Combination With Ramipril Global End Point Trial (ONTARGET). Circulation 2012;126(8):934–41.

81. Rubbo B, Fitzpatrick NK, Denaxas S, et al. Use of electronic health records to ascertain, validate and phenotype acute myocardial infarction: A systematic review and recommendations. Int J Cardiol 2015;187:705–11.

82. Khan NF, Harrison SE, Rose PW. Validity of diagnostic coding within the General Practice Research Database: a systematic review. Br J Gen Pract 2010;60(572):e128–36.

83. Herrett E, Gallagher AM, Bhaskaran K, et al. Data Resource Profile: Clinical Practice Research Datalink (CPRD). Int J Epidemiol 2015;44(3):827–36.

84. Mathur R, Bhaskaran K, Chaturvedi N, et al. Completeness and usability of ethnicity data in UK-based primary care and hospital databases. J Public Health 2014;36(4):684–92.

85. George J, Rapsomaniki E, Pujades-Rodriguez M, et al. How Does Cardiovascular Disease First Present in Women and Men?: Incidence of 12 Cardiovascular Diseases in a Contemporary Cohort of 1 937 360 People. Circulation 2015;132(14):1320–8.

86. Shah AD, Langenberg C, Rapsomaniki E, et al. Type 2 diabetes and incidence of cardiovascular diseases: a cohort study in 1·9 million people. Lancet Diabetes Endocrinol 3(2):105–13.

87. Pujades-Rodriguez M, Timmis A, Stogiannis D, et al. Socioeconomic Deprivation and the Incidence of 12 Cardiovascular Diseases in 1.9 Million Women and Men: Implications for Risk Prediction and Prevention. PLoS ONE 2014;9(8):e104671.

88. Pujades-Rodriguez M, George J, Shah AD, et al. Heterogeneous associations between smoking and a wide range of initial presentations of cardiovascular disease in 1 937 360 people in England: lifetime risks and implications for risk prediction. Int J Epidemiol 2014;44(1):129–41.

89. Rapsomaniki E, Timmis A, George J, et al. Blood pressure and incidence of twelve cardiovascular diseases: lifetime risks, healthy life-years lost, and age-specific associations in 1·25 million people. The Lancet 2014;383(9932):1899–911.

90. White IR, Royston P. Imputing missing covariate values for the Cox model. Stat Med 2009;28(15):1982–98.

91. Rubin DB. Multiple Imputation for Nonresponse in Surveys. New York, USA: Wiley; 1987.

92. Schisterman EF, Cole SR, Platt RW. Overadjustment Bias and Unnecessary Adjustment in Epidemiologic Studies. Epidemiology 2009;20(4):488–95.

93. Rehm J, Irving H, Ye Y, Kerr WC, Bond J, Greenfield TK. Are Lifetime Abstainers the Best Control Group in Alcohol Epidemiology? On the Stability and Validity of Reported Lifetime Abstention. Am J Epidemiol 2008;168(8):866–71.
